# Nonparametric analysis of nonhomogeneous multistate processes with clustered observations

**DOI:** 10.1111/biom.13327

**Published:** 2020-07-21

**Authors:** Giorgos Bakoyannis

**Affiliations:** Department of Biostatistics, Indiana University, Indiana

**Keywords:** multistate model, multicenter, nonparametric test, state occupation probability, transition probability

## Abstract

Frequently, clinical trials and observational studies involve complex event history data with multiple events. When the observations are independent, the analysis of such studies can be based on standard methods for multistate models. However, the independence assumption is often violated, such as in multicenter studies, which makes standard methods improper. This work addresses the issue of nonparametric estimation and two-sample testing for the population-averaged transition and state occupation probabilities under general multistate models with cluster-correlated, right-censored, and/or left-truncated observations. The proposed methods do not impose assumptions regarding the within-cluster dependence, allow for informative cluster size, and are applicable to both Markov and non-Markov processes. Using empirical process theory, the estimators are shown to be uniformly consistent and to converge weakly to tight Gaussian processes. Closed-form variance estimators are derived, rigorous methodology for the calculation of simultaneous confidence bands is proposed, and the asymptotic properties of the nonparametric tests are established. Furthermore, I provide theoretical arguments for the validity of the nonparametric cluster bootstrap, which can be readily implemented in practice regardless of how complex the underlying multistate model is. Simulation studies show that the performance of the proposed methods is good, and that methods that ignore the within-cluster dependence can lead to invalid inferences. Finally, the methods are illustrated using data from a multicenter randomized controlled trial.

## 1 | INTRODUCTION

Frequently, clinical trials and observational studies involve complex multistate event histories. An example is cancer clinical trials where patient event histories typically involve three or more clinical states, such as “cancer-free,” “cancer,” and “death.” Another example is observational studies on coronavirus disease 2019 (COVID-19) progression. In such studies, patients may be hospitalized, then placed to an intensive care unit, on a ventilator, be discharged from the hospital, or die. With independent observations, nonparametric estimation of the transition probabilities for such multistate processes can be performed using the Aalen-Johansen estimator (Aalen and Johansen, 1978). Calculation of confidence bands and nonparametric two-sample tests can be performed using the approaches by Bluhmki et al. (2018) and Bakoyannis (2020), respectively.

The independent observations assumption is often violated in medical research. This is typical in multicenter studies, where the events of individuals within the same center are expected to be associated. Such a multicenter study was the European Organization for Research and Treatment of Cancer (EORTC) trial 10854, which evaluated the effectiveness of the combination of surgery with polychemotherapy compared to surgery alone as a treatment for early breast cancer, and involved 15 hospitals (ie, centers/clusters). Another example is studies involving multiple family members. For example, in a study of COVID-19 progression, members of the same family are expected to have correlated outcomes. When the observations exhibit within-cluster dependence, the traditional Greenwood standard error estimators for the transition probabilities, the simultaneous confidence bands by Bluhmki et al. (2018), and the nonparametric tests by Bakoyannis (2020) are not valid.

Several parametric methods have been proposed for the analysis of multistate models based on clustered observations (Cook et al., 2004; Li and Zhang, 2015; Yiu et al., 2018). However, these methods impose strong parametric assumptions about the underlying multistate processes that are expected to be violated in practice. Chen and Zhou (2013) proposed a semiparametric random-effects approach for cluster-specific inference about nonhomogeneous Markov processes. This approach, which also allows for nonignorable missingness, utilizes a Monte Carlo Expectation Maximization (MCEM) algorithm. Recently, O’Keeffe et al. (2018) proposed a nonparametric approach for cluster-specific inference based on correlated observations from a general multistate model. This approach, similar to the Chen and Zhou (2013) method, accounts for the within-cluster dependence by incorporating random effects. Estimation in this case relies on numerical integration. There are no other nonparametric approaches for clustered multistate data that utilize random effects that I am aware of. The current semiparametric and nonparametric proposals for clustered observations that utilize random effects (Chen and Zhou, 2013; O’Keeffe et al., 2018) have several limitations. First, they impose strong parametric assumptions on the random effects. Also, these random effects introduce only a restrictive positive within-cluster association. Second, they tend to be computationally intensive, which may restrict their use with larger data sets. Third, they do not establish the asymptotic properties of the proposed estimators for the transition probabilities. Moreover, they do not provide methodology for confidence bands and nonparametric hypothesis testing. Fourth, they do not consider the case of informative cluster size (ICS), where there is an association between cluster size and observed events. Finally, in many applications, population-averaged inference is more scientifically relevant than cluster-specific inference. This is the case with the EORTC trial 10854. To our knowledge, only Lan et al. (2017) proposed a method for nonparametric population-averaged inference about state occupation probabilities in general multistate models, allowing for ICS. However, this approach is for current status data and not the usual right-censored or left-truncated multistate data. Moreover, the asymptotic properties of this method have not been established, and there is no methodology for confidence bands and nonparametric tests.

To the best of my knowledge, the issue of nonparametric population-averaged inference for event probabilities in general multistate models with cluster-correlated, right-censored, and/or left-truncated observations has not been addressed so far. In this work, I address this issue by proposing rigorous estimators and methodology for standard error estimation, simultaneous confidence bands, and nonparametric two-sample Kolmogorov-Smirnov–type tests. The asymptotic properties of the proposed methods are rigorously established using modern empirical process theory and closed-form variance estimators are provided. In addition, I establish the validity of the nonparametric cluster bootstrap and show how it can be used for the calculation of simultaneous confidence bands and *P*-values. This is particularly useful in practice, since it provides a convenient way to conduct inference using off-the-shelf software. The proposed methods do not impose restrictive parametric assumptions or assumptions regarding the within-cluster dependence. I additionally allow for ICS and nonhomogeneous processes that are non-Markov. Simulation studies show that the methods perform well and that standard methods for independent observations provide severely under-estimated standard errors and confidence bands with a poor coverage rate. Finally, the methods are illustrated using data from the multicenter EORTC trial 10854.

## 2 | NONPARAMETRIC INFERENCE

### 2.1 | Nonhomogeneous Markov processes

Consider a Markov multistate process {*X*(*t*) : *t* ∈ [0, *τ*]}, for some *τ* < ∞, with a finite set of states S={1,…,k} and a subset T⊂S that includes the possible absorbing states (eg, death). For situations without absorbing states set T=∅. The Markov assumption will be relaxed later in Section 2.6. Let ${\overset{ˇ}{N}}_{hj}(t)$ be the number of direct transitions from state *h* to state *j*, for *h* ≠ *j*, which occurred by time *t* (in the absence of right censoring and left truncation). Also, let ${\overset{ˇ}{Y}}_{h}(t)$ be the at-risk process for state *h*, with ${\overset{ˇ}{Y}}_{h}(t)=1$ if the process is at state *h* just before time *t*, and ${\overset{ˇ}{Y}}_{h}(t)=0$ otherwise. A key quantity of interest is the transition probability which is defined as ${\tilde{P}}_{0,hj}(s,t)=\text{Pr}(X(t)=j|X(s)=h,F_{s^{{}_{-}}})=\text{Pr}(X(t)=j|X(s)=h)$, h,j∈S, 0 ≤ *s* < *t* ≤ *τ*, where $F_{s^{{}_{-}}}=\sigma \langle \{{\overset{ˇ}{N}}_{hj}(u)\;:0\leq u<s,h\neq j\}\rangle$ is the event history prior to time *s*. The subindex 0 is used to indicate the true (unknown) parameter value. Note that the conditional independence from the prior history $F_{s^{{}_{-}}}$ above is the Markov assumption. Another key quantity is the cumulative transition intensity which is defined as ${\tilde{A}}_{0,hj}(t)=\int_{0}^{t}\frac{dE{\overset{ˇ}{N}}_{hj}(u)}{E{\overset{ˇ}{Y}}_{h}(u)},\;h\neq j,\;t\in [0,\tau ]$, with ${\tilde{A}}_{0,hh}(t)=-\sum_{j\neq h}{\tilde{A}}_{0,hj}(t)$, by the Kolmogorov forward equation (Aalen et al., 2008). The *k* × *k* matrix ${\tilde{P}}_{0}(s,t)$, 0 ≤ *s* < *t* ≤ *τ*, of transition probabilities can be defined based on the *k* × *k* matrix ${\tilde{A}}_{0}(t)$ of cumulative transition intensities as ${\tilde{P}}_{0}(s,t)=\Pi_{\left(s,t\right]}\{I_{k}+d{\tilde{A}}_{0}(u)\}$, where Π is the product integral and **I**_*k*_ is the *k* × *k* identity matrix. Finally, the state occupation probability is defined as ${\tilde{P}}_{0,j}(t)=\text{Pr}(X(t)=j)=\sum_{h\in T^{c}}{\tilde{P}}_{0,h}(0){\tilde{P}}_{0,hj}(0,t)$, j∈S, *t* ∈ [0, *τ*].

### 2.2 | Clustered observations

Suppose that a study involves *n* clusters of observations of the Markov process {*X*(*t*) : *t* ∈ [0, *τ*]}, with *M_i_* observations in the *i*th cluster. The observable data are the possibly right-censored and/or left-truncated counting processes {*N*_*im*,*hj*_(*t*) : *h* ≠ *j*, *t* ∈ [0, *τ*]} and at-risk processes $\left\{Y_{im,h}(t)\;:h\in T^{c},t\in [0,\tau ]\right\}$, for *i* = 1, … , *n* and *m* = 1, … , *M_i_*. The process *N*_*im*,*hj*_(*t*) represents the number of *observed* direct transitions from state *h* to state *j*, *h* ≠ *j*, in [0, *t*] (which occurred after the left truncation time and prior to the right censoring time), for the *m*th observation in the *i*th cluster. The process *Y*_*im*,*h*_(*t*) is equal to 1 if the *m*th observation in the *i*th cluster is at state *h and* under observation just before time *t*, and *Y*_*im*,*h*_(*t*) = 0 otherwise. The corresponding *complete* (ie, not right-censored and not left-truncated) counterparts are denoted as ${\overset{ˇ}{N}}_{im,hj}(t)$ and ${\overset{ˇ}{Y}}_{im,h}(t)$. The processes $\left\{\sum_{m=1}^{M_{i}}N_{im,hj}(t)\;:h\neq j,t\in [0,\tau ]\right\}$ and $\left\{\sum_{m=1}^{M_{i}}Y_{im,h}(t)\;:h\in T^{c},t\in [0,\tau ]\right\}$ are assumed i.i.d. across clusters. However, an arbitrary within-cluster dependence for the individual observations is allowed. In this article, it is assumed that the cluster sizes *M_i_*, *i* = 1, … , *n* are either constant or i.i.d. random positive integers. Furthermore, for the latter case, the counting and at-risk processes are allowed to depend on cluster size *M_i_* (informative or nonignorable cluster size). For the sake of generality, *M_i_* is treated as random and informative in this article. However, the methods presented here are trivially applicable to simpler situations where cluster size *M_i_* is either noninformative or fixed. The right censoring and left truncation times are assumed to be independent of both multistate process of interest and cluster size *M_i_*. Also, the main i.i.d. observations assumption implies that, marginally, censoring and truncations times are i.i.d. across clusters. However, between-cluster heterogeneity (eg, different hospitals can have different censoring distributions, conditionally on some hospital-specific random effect) and an arbitrary within-cluster dependence are allowed for censoring and truncation.

When cluster size is random and informative, there are typically two populations of interest (Seaman et al., 2014). The first one is the population of *all cluster members* (ACM), eg, the population of all teeth in dental studies or the population of all patients in multicenter studies. Larger clusters are overrepresented in this population. The second is the population of *typical cluster members* (TCM). This population is formed by selecting one representative member from each cluster (eg, a typical tooth from each patient in dental studies or a typical patient from each center in multicenter studies). Thus, each cluster is equally represented in this population. The population-averaged state occupation probabilities over the ACM population are defined, similar to marginal generalized linear models (Seaman et al., 2014), as $P_{0,j}(t)=\frac{E\{M_{1}I(X_{1m}(t)=j)\}}{{\mathrm{EM}}_{1}}$, j∈S, *t* ∈ [0, *τ*], for a randomly selected cluster member *m.* These can be seen as weighted averages where larger clusters have a larger influence on these probabilities. The population-averaged state occupation probabilities over the TCM population are defined as $P_{0,j}^{\prime }(t)=EI(X_{1m}(t)=j)$, j∈S, for a randomly selected cluster member *m*. In this case, all clusters contribute a single (randomly selected) member and, therefore, all clusters have the same weight on the resulting probabilities. The two versions of population-averaged transition probabilities can be defined similarly. This leads to the population-averaged cumulative transition intensities $A_{0,hj}(t)=\int_{0}^{t}\frac{dE\{M_{1}{\overset{ˇ}{N}}_{1m,hj}(u)\}}{E\{M_{1}{\overset{ˇ}{Y}}_{1m,h}(u)\}}$, *h* ≠ *j*, with $A_{0,hh}(t)=-\sum_{j\neq h}A_{0,hj}(t)$, and $A_{0,hj}^{\prime }(t)=\int_{0}^{t}\frac{dE{\overset{ˇ}{N}}_{1m,hj}(u)}{E{\overset{ˇ}{Y}}_{1m,h}(u)}$, *h* ≠ *j*, with $A_{0,hh}^{\prime }(t)=-\sum_{j\neq h}A_{0,hj}^{\prime }(t)$. Based on the corresponding matrices **A**_0_(*t*) and $A_{0}^{\prime }(t)$, the population-averaged transition probability matrices can be expressed as the product integrals (by the Kolmogorov forward equations) $P_{0}(s,t)=\Pi_{\left(s,t\right]}\{I_{k}+dA_{0}(u)\}$ and $P_{0}^{\prime }(s,t)=\Pi_{\left(s,t\right]}\{I_{k}+dA_{0}^{\prime }(u)\},0\leq s\leq t\leq \tau$. If cluster size is either noninformative or constant then $P_{0}=P_{0}^{\prime }$ and $P_{0,j}=P_{0,j}^{\prime }$, for j∈S. However, if cluster size is informative, it is expected that $P_{0}\neq P_{0}^{\prime }$ and $P_{0,j}\neq P_{0,j}^{\prime }$, j∈S. If the probability of a particular event over the ACM population is higher (lower) than the probability of that event over the TCM population, then the proportion of this event is larger (smaller) in larger clusters. This is because a population-averaged probability over the ACM population is dominated by larger clusters under ICS. Depending on the setting, the difference between the two probabilities may be attributed to systematic differences in important individuals’ characteristics between larger and smaller clusters of observations. For example, in multicenter studies, patients with more advanced disease, and thus more prone to poor health outcomes, may tend to choose (or be advised to attend) larger clinics. When clusters are health care facilities or providers, another reason for the difference between the two population-averaged probabilities may be systematic differences in the performance of facilities or providers with more patients.

In the EORTC10854 trial, the population-averaged probabilities of cancer and death over the ACM population provide information about the effectiveness of the combined intervention on a *typical patient* from the population of all patients. In these probabilities, hospitals with more patients are naturally overweighted as they account for a larger portion of patients in the population. On the other hand, the population-averaged probabilities over the TCM population provide information about the effectiveness of the combined intervention on a typical patient from a *typical hospital setting.* These probabilities weight each hospital equally and, thus, they are not dominated by hospitals with more patients, which may have different performance and/or patient characteristics compared to those with less patients. Thus, they provide information about effectiveness on a typical patient from an average performing hospital.

### 2.3 | Estimation of transition probabilities

To nonparametrically estimate the population-averaged transition probability matrices **P**_0_ and $P_{0}^{\prime }$, we first estimate the population-averaged cumulative transition intensity matrices **A**_0_ and $A_{0}^{\prime }$, and then utilize the relationships $P_{0}(s,t)=\Pi_{\left(s,t\right]}\{I_{k}+dA_{0}(u)\}$ and $P_{0}^{\prime }(s,t)=\Pi_{\left(s,t\right]}\{I_{k}+dA_{0}^{\prime }(u)\},\;0\;\leq s\;\leq \;t\;\leq \;\tau$. Let $N_{i\cdot ,hj}(t)\equiv \sum_{m=1}^{M_{i}}N_{im,hj}(t)$, for *h* ≠ *j*, and $Y_{i\cdot ,h}(t)\equiv \sum_{m=1}^{M_{i}}Y_{im,h}(t)$, for $h\in T^{c}$. In Web Appendix B.2, it is shown that $A_{0,hj}(t)=\int_{0}^{t}\frac{dEN_{1\cdot ,hj}(u)}{EY_{1\cdot ,h}(u)}$, *h* ≠ *j*. Therefore, a natural estimator of *A*_0,*hj*_(*t*) is ${\hat{A}}_{n,hj}(t)=\int_{0}^{t}\frac{d\{\sum_{i=1}^{n}N_{i\cdot ,hj}(u)\}}{\sum_{i=1}^{n}Y_{i\cdot ,h}(u)}$, *h* ≠ *j*, *t* ∈ [0, *τ*]. Similar arguments lead to the conclusion that $A_{0,hj}^{\prime }(t)=\int_{0}^{t}\frac{dE\{M_{i}^{-1}N_{i\cdot ,hj}(u)\}}{E\{M_{i}^{-1}Y_{i\cdot ,h}(u)\}}$, *h* ≠ *j*, and thus a natural nonparametric estimator of $A_{0,hj}^{\prime }(t)$ is ${\hat{A}}_{n,hj}^{\prime }(t)=\int_{0}^{t}\frac{d\{\sum_{i=1}^{n}M_{i}^{-1}N_{i\cdot ,hj}(u)\}}{\sum_{i=1}^{n}M_{i}^{-1}Y_{i\cdot ,h}(u)}$, *h* ≠ *j*, *t* ∈ [0, *τ*]. Then, the proposed plug-in estimators of **P**_0_ and $P_{0}^{\prime }$ are ${\hat{P}}_{n}(s,t)=\Pi_{\left(s,t\right]}\{I_{k}+d{\hat{A}}_{n}(u)\}$ and ${\hat{P}}_{n}^{\prime }(s,t)=\Pi_{\left(s,t\right]}\{I_{k}+d{\hat{A}}_{n}^{\prime }(u)\}$, where ${\hat{A}}_{n}(t)$ and ${\hat{A}}_{n}^{\prime }(t)$ are the *k* × *k* matrices with off-diagonal elements ${\hat{A}}_{n,hj}(t)$ and ${\hat{A}}_{n,hj}^{\prime }(t)$, and diagonal elements $-\sum_{j\neq h}{\hat{A}}_{n,hj}(t)$ and $-\sum_{j\neq h}{\hat{A}}_{n,hj}^{\prime }(t)$, *h* = 1, … , *k*, respectively. In the special case with fixed cluster size, ${\hat{P}}_{n}={\hat{P}}_{n}^{\prime }$. The estimator ${\hat{P}}_{n}$ can be seen as the working independence Aalen-Johansen estimator. We call ${\hat{P}}_{n}^{\prime }$ the weighted by cluster size working independence Aalen-Johansen estimator. The following theorem states the uniform consistency of ${\hat{P}}_{n}$ and ${\hat{P}}_{n}^{\prime }$.

**Theorem 1.** *Suppose that conditions C1 to C5 in* Web Appendix B.1 *hold and define the norm* ||**A**|| = sup_*l*_ ∑_*r*_ |*a_lr_*| *for some matrix* **A** = [*a_lr_*]. *Then, for any s* ∈ [0, *τ*], *as n* → ∞

$$\underset{t\in [s,\tau ]}{\sup}\|{\hat{P}}_{n}(s,t)-P_{0}(s,t)\|\overset{\mathrm{as}*}{\to }0\;\mathrm{and}\;\underset{t\in [s,\tau ]}{\sup}\|{\hat{P}}_{n}^{\prime }(s,t)-P_{0}^{\prime }(s,t)\|\overset{\mathrm{as}*}{\to }0.$$

The proof of Theorem 1 can be found in Web Appendix B.2. Note that, even though the standard Aalen-Johansen estimator is consistent for **P**_0_, the usual standard error estimators are invalid with clustered data as they ignore the within-cluster dependence.

Next, the asymptotic distributions of the estimators are studied. Let *γ_ihj_*(*s*, *t*) and $\gamma_{ihj}^{\prime }(s,t)$ denote the influence functions of the estimators ${\hat{P}}_{n,hj}(s,t)$ and ${\hat{P}}_{n,hj}^{\prime }(s,t),\;0\leq s\leq t\leq \tau$, respectively. Explicit formulas for the influence functions are provided in Web Appendix A. Next, define the estimated process ${\hat{B}}_{n,hj}(s,\cdot )=n^{-1∕2}\sum_{i=1}^{n}{\hat{\gamma }}_{ihj}(s,\cdot )\xi_{i}$, for $h\in T^{c}$ and j∈S, where *ξ_i_*, *i* = 1, … , *n*, are i.i.d. standard normal random variables, and ${\hat{\gamma }}_{ihj}(s,\cdot )$ is the estimated version of *γ_ihj_*(*s*, ⋅) (see Web Appendix A for details). Similarly, define the estimated process ${\hat{B}}_{n,hj}^{\prime }(s,\cdot )=n^{-1∕2}\sum_{i=1}^{n}{\hat{\gamma }}_{ihj}^{\prime }(s,\cdot )\xi_{i}$, for $h\in T^{c}$ and j∈S. These estimated processes will be used for the calculation of simultaneous confidence bands. An alternative method for inference is the nonparametric cluster bootstrap. Calculation of a bootstrap version of ${\hat{P}}_{n}$ and ${\hat{P}}_{n}^{\prime }$, denoted by ${\hat{P}}_{n}^{*}$ and ${\hat{P}}_{n}^{\prime *}$, respectively, can be easily performed by randomly sampling *n* clusters with replacement from the original data set, and then calculating the proposed estimators based on the resulting bootstrap data set.

**Theorem 2.** *Suppose that conditions C1 to C6 in* Web Appendix B.1 *hold*. *Then, for any* $h\in T^{c}$, j∈S, *and s* ∈ [0, *τ*),

- $\sqrt{n}\{{\hat{P}}_{n,hj}(s,t)-P_{0,hj}(s,t)\}=n^{-1∕2}\sum_{i=1}^{n}\gamma_{ihj}(s,t)+o_{p}(1)$ *and* $\sqrt{n}\{{\hat{P}}_{n,hj}^{\prime }(s,t)-P_{0,hj}^{\prime }(s,t)\}=n^{-1∕2}\sum_{i=1}^{n}\gamma_{ihj}^{\prime }(s,t)+o_{p}(1),\;t\in [s,\tau ]$. *Moreover, the classes of functions* {*γ_ihj_*(*s*, *t*) : *t* ∈ [*s*, *τ*]} *and* $\left\{\gamma_{ihj}^{\prime }(s,t)\;:\;t\in [s,\tau ]\right\}$ *are P-Donsker;*
- ${\hat{B}}_{hj}(s,\cdot )⇝G_{hj}(s,\cdot )$ *and* $\sqrt{n}\{{\hat{P}}_{n,hj}^{*}(s,\cdot )-{\hat{P}}_{n,hj}(s,\cdot )\}⇝G_{hj}(s,\cdot )$ *in D*[*s*, *τ*], *conditionally on the observed data, where* $G_{hj}(s,\cdot )$ *is the limiting process of* $\sqrt{n}\{{\hat{P}}_{n,hj}(s,\cdot )-P_{0,hj}(s,\cdot )\}$*;*
- ${\hat{B}}_{hj}^{\prime }(s,\cdot )⇝G_{hj}^{\prime }(s,\cdot )$ *and* $\sqrt{n}\{{\hat{P}}_{n,hj}^{\prime *}(s,\cdot )-{\hat{P}}_{n,hj}^{\prime }(s,\cdot )\}⇝G_{hj}^{\prime }(s,\cdot )$ *in D*[*s*, *τ*], *conditionally on the observed data, where* $G_{hj}^{\prime }(s,\cdot )$ *is the limiting process of* $\sqrt{n}\{{\hat{P}}_{n,hj}^{\prime }(s,\cdot )-P_{0,hj}^{\prime }(s,\cdot )\}$.

The proof of Theorem 2 can be found in Web Appendix B.3. In Web Appendix B.5, condition C6 is relaxed. By Theorem 2, $\sqrt{n}\{{\hat{P}}_{n,hj}(s,\cdot )-P_{0,hj}(s,\cdot )\}$ and $\sqrt{n}\{{\hat{P}}_{n,hj}(s,\cdot )-P_{0,hj}(s,\cdot )\}$ converge weakly to the mean-zero Gaussian processes $G_{hj}(s,\cdot )$ and $G_{hj}^{\prime }(s,\cdot )$, respectively. The covariance functions of $G_{hj}(s,\cdot )$ and $G_{hj}^{\prime }(s,\cdot )$ at the time points *t*_1_ and *t*_2_ are *E*{*γ*_1*hj*_(*s*, *t*_1_)*γ*_1*hj*_(*s*, *t*_2_)} and $E\{\gamma_{1hj}^{\prime }(s,t_{1})\gamma_{1hj}^{\prime }(s,t_{2})\}$. These covariance functions can be consistently estimated by $n^{-1}\sum_{i=1}^{n}{\hat{\gamma }}_{ihj}(s,t_{1}){\hat{\gamma }}_{ihj}(s,t_{2})$ and $n^{-1}\sum_{i=1}^{n}{\hat{\gamma }}_{ihj}^{\prime }(s,t_{1}){\hat{\gamma }}_{ihj}^{\prime }(s,t_{2})$, respectively. Theorem 2 also implies that the asymptotic distributions of the estimators can be easily approximated by generating realizations of the processes ${\hat{B}}_{hj}(s,\cdot )$ and ${\hat{B}}_{hj}^{\prime }(s,\cdot )$ (through simulating a large number of sets of standard normal variates $\{\xi_{i}{\}}_{i=1}^{n}$) or by cluster bootstrap realizations $\sqrt{n}\{{\hat{P}}_{n,hj}^{*}(s,\cdot )-{\hat{P}}_{n,hj}(s,\cdot )\}$ and $\sqrt{n}\{{\hat{P}}_{n,hj}^{\prime *}(s,\cdot )-{\hat{P}}_{n,hj}^{\prime }(s,\cdot )\}$.

These results can be used for the calculation of pointwise confidence intervals and simultaneous confidence bands. For these procedures consider a differentiable transformation *g*, such as *g*(*x*) = log{−log(*x*)}, to ensure that the limits of the confidence interval and the confidence band lie in the interval (0,1). For the calculation of confidence bands for *P*_0,*hj*_(*s*, ⋅), $h\in T^{c}$, j∈S, and *s* ∈ [0, *τ*), it is useful to consider a weight function ${\hat{q}}_{hj}(s,t)$ that converges uniformly (in probability) to a bounded nonnegative function on an interval [*t*_1_, *t*_2_] ⊂ [*s*, *τ*]. A choice is ${\hat{q}}_{hj}(s,t)=\{1+n^{-1}\sum_{i=1}^{n}{\hat{\gamma }}_{1hj}(s,t{)}^{2}{\}}^{-1}$, where, as argued above, $n^{-1}\sum_{i=1}^{n}{\hat{\gamma }}_{1hj}(s,\cdot {)}^{2}$ is consistent for the true asymptotic variance of $\sqrt{n}\{{\hat{P}}_{n,hj}(s,\cdot )-P_{0,hj}(s,\cdot )\}$. By Theorem 2, the functional delta method, and the continuous mapping theorem it follows that ${\text{sup}}_{t\in [t_{1},t_{2}]}\;|\sqrt{n}{\hat{q}}_{hj}(s,t)\{g({\hat{P}}_{n,hj}(s,t))-g(P_{0,hj}(s,t))\}|$ and ${\text{sup}}_{t\in [t_{1},t_{2}]}\;|{\hat{q}}_{hj}(s,t)\dot{g}({\hat{P}}_{n,hj}(s,t))\sqrt{n}\{{\hat{P}}_{n,hj}(s,t))-P_{0,hj}(s,t)\}|$, have the same asymptotic distribution. The 1 − *α* percentile of this distribution, denoted by *c_α_*, can be estimated as the sample percentile ${\hat{c}}_{\alpha }$ of a large number of simulation realizations of the process ${\text{sup}}_{t\in [t_{1},t_{2}]}\;|{\hat{q}}_{hj}(s,t)\dot{g}({\hat{P}}_{n,hj}(s,t)){\hat{B}}_{hj}(s,t)|$. Alternatively, one can use cluster bootstrap realizations ${\text{sup}}_{t\in [t_{1},t_{2}]}\;|{\hat{q}}_{hj}(s,t)\dot{g}({\hat{P}}_{n,hj}(s,t))\sqrt{n}\{{\hat{P}}_{n,hj}^{*}(s,t)-{\hat{P}}_{n,hj}(s,t)\}|$. Based on this ${\hat{c}}_{\alpha }$, a 1 − *α* simultaneous confidence band can be calculated as $g^{-1}\{g({\hat{P}}_{n,hj}(s,t))\pm \frac{{\hat{c}}_{\alpha }}{\sqrt{n}{\hat{q}}_{hj}(s,t)}\}$, *t* ∈ [*t*_1_, *t*_2_]. In general, simultaneous confidence bands can be unstable toward the earlier or later times of the observation interval (Nair, 1984). To avoid this issue in practice it is suggested to restrict the domain of the confidence band to a set with limits the 10th and 90th or the 5th and 95th percentile of the distribution of transition times from state *h* to state *j*. Calculation of confidence bands for $P_{0,hj}^{\prime }(s,\cdot )$ can be performed similarly.

### 2.4 | Estimation of state occupation probabilities

Natural estimators of the state occupation probabilities *P*_0,*j*_(*t*) and $P_{0,j}^{\prime }(t)$ are ${\hat{P}}_{n,j}(t)=\sum_{h\in T^{c}}\{\frac{\sum_{i=1}^{n}Y_{i\cdot ,h}(0+)}{{\hat{\pi }}_{n}\sum_{i=1}^{n}M_{i}}\}{\hat{P}}_{n,hj}(0,t)$, j∈S, where ${\hat{\pi }}_{n}=n^{-1}\sum_{i=1}^{n}M_{i}^{-1}\sum_{h\in T^{c}}Y_{i\cdot ,h}(0+)$, and ${\hat{P}}_{n,j}^{\prime }(t)=\sum_{h\in T^{c}}\{\frac{\sum_{i=1}^{n}M_{i}^{-1}Y_{i\cdot ,h}(0+)}{n{\hat{\pi }}_{n}}\}\;{\hat{P}}_{n,hj}^{\prime }(0,t)$, j∈S. In these estimators, ${\hat{\pi }}_{n}$ is a consistent estimator of the probability of being under observation at time *t* = 0, denoted as *π*_0_. Here, it is also assumed that *π*_0_ > 0. In the absence of left truncation ${\hat{\pi }}_{n}=\pi_{0}=1$. In the special case with fixed cluster size, ${\hat{P}}_{n,j}={\hat{P}}_{n,j}^{\prime }$, j∈S. Based on Theorem 1, it can be easily shown that ${\hat{P}}_{n,j}(t)$ and ${\hat{P}}_{n,j}^{\prime }(t)$ are uniformly consistent.

In light of Theorem 2, the state occupation probability estimators are asymptotically linear of the form $\sqrt{n}\{{\hat{P}}_{n,j}(t)-P_{0,j}(t)\}=\frac{1}{\sqrt{n}}\sum_{i=1}^{n}\psi_{ij}(t)+o_{p}(1),\;j\in S,\;t\in [0,\tau ]$ and $\sqrt{n}\{{\hat{P}}_{n,j}^{\prime }(t)-P_{0,j}^{\prime }(t)\}=\frac{1}{\sqrt{n}}\sum_{i=1}^{n}\psi_{ij}^{\prime }(t)+o_{p}(1),\;j\in S,\;t\in [0,\tau ]$, where the influence functions *ψ_ij_*(*t*) and $\psi_{ij}^{\prime }(t)$ are provided in Web Appendix A. It follows that, $\sqrt{n}({\hat{P}}_{n,j}-P_{0,j})$ and $\sqrt{n}({\hat{P}}_{n,j}^{\prime }-P_{0,j}^{\prime })$ converge weakly to zero-mean Gaussian processes, with covariance functions *E*{*ψ_ij_*(*t*_1_)*ψ_ij_*(*t*_2_)} and $E\{\psi_{ij}^{\prime }(t_{1})\psi_{ij}^{\prime }(t_{2})\}$, for *t*_1_, *t*_2_ ∈ [0, *τ*]. As with the case of transition probabilities, the estimated influence functions can be used to consistently estimate these covariance functions. Moreover, the estimated processes $n^{-1∕2}\sum_{i=1}^{n}{\hat{\psi }}_{ij}(\cdot )\xi_{i}$ and $n^{-1∕2}\sum_{i=1}^{n}{\hat{\psi }}_{ij}^{\prime }(\cdot )\xi_{i}$ and the cluster bootstrap processes $\sqrt{n}({\hat{P}}_{n,j}^{*}-{\hat{P}}_{n,j})$ and $\sqrt{n}({\hat{P}}_{n,j}^{\prime *}-{\hat{P}}_{n,j}^{\prime })$ can be used to calculate confidence bands, as described for the transition probabilities.

### 2.5 | Two-sample Kolmogorov-Smirnov–type tests

In many settings, the scientific interest is on comparing the transition probabilities for a particular transition, or the state occupation probabilities for a particular state, between two groups, say groups 1 and 2. For example, consider a multicenter randomized controlled trial where the goal is to assess whether the probability of cancer relapse differs between those receiving an experimental treatment and those receiving a control treatment. Depending on what is the most relevant population-averaged quantity for the given context, the null hypothesis in terms of the transition probability is either *H*_0_ : *P*_0,1*hj*_(*s*, ⋅) = *P*_0,2*hj*_(*s*, ⋅) or $H_{0}\;:\;P_{0,1hj}^{\prime }(s,\cdot )=P_{0,2hj}^{\prime }(s,\cdot )$, for some *s* ∈ [0, *τ*). In terms of the state occupation probability, the null hypothesis is either *H*_0_ : *P*_0,1*j*_ = *P*_0,2*j*_ or $H_{0}\;:\;P_{0,1j}^{\prime }=P_{0,2j}^{\prime }$. Let *M*_1*i*_ and *M*_2*i*_ be the number of observations from the *i*th cluster, which belong to groups 1 and 2, respectively, with *M*_1*i*_ + *M*_2*i*_ = *M_i_*, *i* = 1, … , *n*. Here, the situation where min(*M*_1*i*_, *M*_2*i*_) > 0 is considered, that is each cluster contains at least one observation from both groups. Finally, let *N*_*ipm*,*hj*_(*t*), *h* ≠ *j*, and *Y*_*ipm*,*h*_(*t*), $h\in T^{c}$ be the counting and at-risk processes for the *m*th observation in the *p*th group in the *i*th cluster.

Based on this setup, define the estimators of the pointwise between-group difference of the transition probabilities as ${\hat{\Delta }}_{n,hj}(s,t)=\{{\hat{P}}_{n,1hj}(s,t)-{\hat{P}}_{n,2hj}(s,t)\}$, *t* ∈ [*s*, *τ*], where ${\hat{P}}_{n,phj}$, *p* = 1, 2, is the estimator of *P*_0,*phj*_ from the *p*th group and ${\hat{\Delta }}_{n,hj}^{\prime }(s,t)=\{{\hat{P}}_{n,1hj}^{\prime }(s,t)-{\hat{P}}_{n,2hj}^{\prime }(s,t)\}$, *t* ∈ [*s*, *τ*], where ${\hat{P}}_{n,phj}^{\prime }$, *p* = 1, 2, is the estimator of $P_{0,phj}^{\prime }$ from the *p*th group, for some *s* ∈ [0, *τ*). Similarly, define the differences between the population-averaged state occupation probabilities as ${\hat{\Delta }}_{n,j}(t)=\{{\hat{P}}_{n,1j}(t)-{\hat{P}}_{n,2j}(t)\}$, *t* ∈ [0, *τ*], where ${\hat{P}}_{n,pj}$, *p* = 1, 2, is the estimator of *P*_0,*pj*_ from the *p*th group, and ${\hat{\Delta }}_{n,j}^{\prime }(t)=\{{\hat{P}}_{n,1j}^{\prime }(t)-{\hat{P}}_{n,2j}^{\prime }(t)\}$, *t* ∈ [0, *τ*], where ${\hat{P}}_{n,pj}^{\prime }$, *p* = 1, 2, is the estimator of $P_{0,pj}^{\prime }$ from the *p*th group. The corresponding nonparametric cluster bootstrap realizations of the above differences are denoted by ${\hat{\Delta }}_{n,hj}^{*}(s,t)$, ${\hat{\Delta }}_{n,hj}^{\prime *}(s,t)$, ${\hat{\Delta }}_{n,j}^{*}(t)$, and ${\hat{\Delta }}_{n,j}^{\prime *}(t)$. It is important to note that these nonparametric cluster bootstrap realizations are generated by randomly sampling *n* clusters with replacement, as described in Sections 2.3. Based on these differences, define the Kolmogorov-Smirnov–type test statistics $K_{n,hj}(s)={\text{sup}}_{t\in [s,\tau ]}\;|{\hat{W}}_{hj}(t){\hat{\Delta }}_{n,hj}(s,t)|$, for some appropriate weight function ${\hat{W}}_{hj}(t)$ and some *s* ∈ [0, *τ*), and $K_{n,j}={\text{sup}}_{t\in [0,\tau ]}\;|{\hat{W}}_{j}(t){\hat{\Delta }}_{n,j}(t)|$. The corresponding tests for ${\hat{\Delta }}_{n,hj}^{\prime }(s,t)$ and ${\hat{\Delta }}_{n,j}^{\prime }(t)$, denoted by $K_{n,hj}^{\prime }(s)$ and $K_{n,j}^{\prime }$, are defined in the same manner. The weights ${\hat{W}}_{hj}(t)$, ${\hat{W}}_{hj}^{\prime }(t)$, ${\hat{W}}_{j}(t)$, and ${\hat{W}}_{j}^{\prime }(t)$ are assumed to be uniformly consistent (in probability) for the nonnegative and uniformly bounded fixed functions *W_hj_*(*t*), $W_{hj}^{\prime }(t)$, *W_j_*(*t*), and $W_{j}^{\prime }(t)$. The importance of the weight functions lies on the fact that they can restrict the comparison interval to a set of times where both groups under comparison have nonzero observations at risk for the transition of interest. An example of such a weight function is ${\hat{W}}_{hj}(t)=I[\prod_{l\in L(h,j)}{\overset{‒}{Y}}_{1,l}(t){\overset{‒}{Y}}_{2,l}(t)>0]$, where L(h,j)={d∈S : *d* is a transient state that can be visited during the transition *h* → *j*} and ${\overset{‒}{Y}}_{p,h}(t)=n_{p}^{-1}\sum_{i=1}^{n_{p}}Y_{pi\cdot ,h}(t)$, for the group *p* = 1, 2, with *Y*_*pi*⋅,*h*_(*t*) denoting the sum of the at-risk process for state *h* in the *i*th cluster and the *p*th group. Similarly, this type of weight can be defined for the state occupation probabilities as ${\hat{W}}_{j}(t)=I[\prod_{l\in \cup_{h\in T^{c}}L(h,j)}{\overset{‒}{Y}}_{1,l}(t){\overset{‒}{Y}}_{2,l}(t)>0]$. The weights ${\hat{W}}_{hj}^{\prime }(t)$ and ${\hat{W}}_{j}^{\prime }(t)$ are defined similarly. The weight functions can also be used to assign less weight to observation times with a smaller number of observations, where the estimated difference tends to be unstable. An example of such weight functions is ${\hat{W}}_{hj}(t)=\frac{\prod_{l\in L(h,j)}{\overset{‒}{Y}}_{1,l}(t){\overset{‒}{Y}}_{2,l}(t)}{\sum_{l\in L(h,j)}\{{\overset{‒}{Y}}_{1,l}(t)+{\overset{‒}{Y}}_{2,l}(t)\}}$ and ${\hat{W}}_{j}(t)=\frac{\prod_{l\in \cup_{h\in T^{c}}L(h,j)}{\overset{‒}{Y}}_{1,l}(t){\overset{‒}{Y}}_{2,l}(t)}{\sum_{l\in \cup_{h\in T^{c}}L(h,j)}\{{\overset{‒}{Y}}_{1,l}(t)+{\overset{‒}{Y}}_{2,l}(t)\}}$. The corresponding weights ${\hat{W}}_{hj}^{\prime }(t)$ and ${\hat{W}}_{j}^{\prime }(t)$ can be similarly defined by replacing ${\overset{‒}{Y}}_{p,h}(t)$ with $n_{p}^{-1}\sum_{i=1}^{n_{p}}M_{pi}^{-1}Y_{\;pi\cdot ,h}(t)$, for the group *p* = 1,2. In practice, the use of this latter type of weight functions is suggested. The calculation of *P*-values can be based on nonparametric cluster bootstrap or the influence functions for the group-specific estimators ${\hat{P}}_{n,phj}(s,t)$ and ${\hat{P}}_{n,pj}(t)$, *p* = 1, 2. These influence functions, denoted by *γ*_*p*,*ihj*_(*s*, *t*) and *ψ*_*p*,*ij*_(*t*), respectively, are provided in Web Appendix A. Now, define the estimated processes ${\hat{C}}_{n,hj}(s,t)={\hat{W}}_{hj}(t)n^{-1∕2}\sum_{i=1}^{n}\{{\hat{\gamma }}_{1,ihj}(s,t)-{\hat{\gamma }}_{2,ihj}(s,t)\}\xi_{i}$, *t* ∈ [*s*, *τ*], for some *s* ∈ [0, *τ*], where *ξ_i_*, are independent standard normal variables and the influence functions are estimated as described in Web Appendix A, and ${\hat{C}}_{n,j}(t)={\hat{W}}_{j}(t)n^{-1∕2}\sum_{i=1}^{n}\{{\hat{\psi }}_{1,ij}(t)-{\hat{\psi }}_{2,ij}(t)\}\xi_{i}$, *t* ∈ [0, *τ*]. Similarly, one can define the estimated processes ${\hat{C}}_{n,hj}(s,t)$ and ${\hat{C}}_{n,j}^{\prime }(t)$ which correspond to the tests for ${\hat{\Delta }}_{n,hj}^{\prime }(s,t)$ and ${\hat{\Delta }}_{n,j}^{\prime }(t)$.

**Theorem 3.** *Suppose that conditions C1, C2, C3′, C4′, C5, and C6′ in* Web Appendix B.1 *hold*. *Then, under the null hypothesis and for any* $h\in T^{c}$, j∈S, *and s* ∈ [0, *τ*),

- $\sqrt{n}{\hat{W}}_{hj}(\cdot ){\hat{\Delta }}_{n,hj}(s,\cdot )⇝Z_{hj}(s,\cdot )$ *in D*[*s*, *τ*], *where* $Z_{hj}(s,\cdot )$ *is a tight zero-mean Gaussian process with covariance function W_hj_*(*t*_1_)*W_hj_*(*t*_2_)*E*[{*γ*_1,1*hj*_(*s*, *t*_1_) − *γ*_2,1*hj*_(*s*, *t*_1_)}{*γ*_1,1*hj*_(*s*, *t*_2_) − *γ*_2,1*hj*_(*s*, *t*_2_)}], *for t*_1_, *t*_2_ ∈ [*s*, *τ*]. *Moreover*, ${\hat{C}}_{n,hj}(s,\cdot )⇝Z_{hj}(s,\cdot )$ *and* $\sqrt{n}{\hat{W}}_{hj}(\cdot )\{{\hat{\Delta }}_{n,hj}^{*}(s,\cdot )-{\hat{\Delta }}_{n,hj}(s,\cdot )\}⇝Z_{hj}(s,\cdot )$ *in D*[*s*, *τ*], *conditionally on the observed data*.
- $\sqrt{n}{\hat{W}}_{hj}{\hat{\Delta }}_{n,j}⇝Z_{j}$ *in D*[0, *τ*], *where* $Z_{j}$ *is a tight zero-mean Gaussian process with covariance function W_j_*(*t*_1_)*W_j_*(*t*_2_)*E*[{*ψ*_1,1*j*_(*s*, *t*_1_) − *ψ*_2,1*j*_(*s*, *t*_1_)}{*ψ*_1,1*j*_(*s*, *t*_2_) − *ψ*_2,1*j*_(*s*, *t*_2_)}], *for t*_1_, *t*_2_ ∈ [*s*, *τ*]. *Moreover*, ${\hat{C}}_{n,j}⇝Z_{j}$ *and* $\sqrt{n}{\hat{W}}_{j}({\hat{\Delta }}_{n,j}^{*}-{\hat{\Delta }}_{n,j})⇝Z_{j}$ *in D*[0, *τ*], *conditionally on the observed data*.

The proof of Theorem 3 can be found in Web Appendix B.4. There, it is also shown that the tests are consistent against any fixed alternative hypothesis. A relaxation of condition C6′ is considered in Web Appendix B.5. It can be easily shown that a similar version of Theorem 3 holds for the differences ${\hat{\Delta }}_{h,hj}^{\prime }(s,\cdot )$ and ${\hat{\Delta }}_{h,j}^{\prime }$. Based on Theorem 3 and the continuous mapping theorem it follows that, under the null hypothesis, $\sqrt{n}K_{n,hj}(s)\;\overset{d}{\to }\;{\text{sup}}_{t\in [s,\tau ]}\;|Z_{hj}(s,t)|$, for any *s* ∈ [0, *τ*), and $\sqrt{n}K_{n,j}\;\overset{d}{\to }\;{\text{sup}}_{t\in [0,\tau ]}\;|Z_{j}(t)|$. These asymptotic null distributions are complicated to use in practice for the calculation of *P*-values. However, by Theorem 3, one can simulate realizations from these null distributions by simulating a sufficiently large number of sets $\{\xi_{i}{\}}_{i=1}^{n}$ of standard normal variables and then calculating samples from these null distributions as ${\text{sup}}_{t\in [s,\tau ]}\;|{\hat{C}}_{n,hj}(s,t)|$ and ${\text{sup}}_{t\in [0,\tau ]}\;|{\hat{C}}_{n,j}(t)|$. Alternatively, one can use a sufficiently large number of nonparametric cluster bootstraps ${\hat{\Delta }}_{n,hj}^{*}(s,t)$, *t* ∈ [*s*, *τ*], and ${\hat{\Delta }}_{n,j}^{*}(t)$, *t* ∈ [0, *τ*] and, then, calculate realizations from the asymptotic null distributions as $\sqrt{n}\;{\text{sup}}_{t\in [s,\tau ]}\;|{\hat{W}}_{hj}(t)\{{\hat{\Delta }}_{n,hj}^{*}(s,t)-{\hat{\Delta }}_{n,hj}(s,t)\}|$ and $\sqrt{n}\;{\text{sup}}_{t\in [0,\tau ]}\;|{\hat{W}}_{j}(t)\{{\hat{\Delta }}_{n,j}^{*}(t)-{\hat{\Delta }}_{n,j}(t)\}|$. The *P*-value can then be estimated as the proportion of these simulation realizations, which are greater than or equal to the actual value of the test statistic based on the observed data.

### 2.6 | Non-Markov processes

When the multistate process *X*(*t*) is non-Markov, the transition probabilities and transition intensities depend on the prior event history $F_{t^{{}_{-}}}$. In this case, the population-averaged transition intensities defined in Section 2.2 are the *partly conditional transition intensities*, which are not conditional on the prior history $F_{t^{{}_{-}}}$. Such marginal intensities have been argued to be meaningful quantities even for non-Markov processes because they describe the marginal (ie, unconditional on the prior history) behavior of the process (Datta and Satten, 2001; Glidden, 2002). With independent observations from a non-Markov process, Datta and Satten (2001) showed that the Nelson-Aalen estimator of the cumulative transition intensities and the Aalen-Johansen estimator of the state occupation probabilities are consistent for the corresponding marginal quantities. Using the same arguments to those presented by Datta and Satten (2001) it can be shown that, with clustered observations from a non-Markov process, the proposed estimators of the (marginal) population-averaged cumulative transition intensities and state occupation probabilities are consistent. Similarly, as in the case with independent observations (Titman, 2015), the proposed estimators ${\hat{P}}_{n}(0,t)$ and ${\hat{P}}_{n}^{\prime }(0,t)$ are consistent for the population-averaged **P**_0_(0, *t*) and $P_{0}^{\prime }(0,t)$ under right censoring, even for non-Markov processes. In the presence of left truncation, consistent estimation requires calculating ${\hat{P}}_{n}(0,t)$ and ${\hat{P}}_{n}^{\prime }(0,t)$ using only the subset of individuals who were under observation at *t* = 0. However, for *s* > 0, the proposed estimators ${\hat{P}}_{n}(s,t)$ and ${\hat{P}}_{n}^{\prime }(s,t)$ are not consistent in general for non-Markov processes, as in the case with independent observations (Titman, 2015). In such cases, following the idea of landmarking by Putter and Spitoni (2018), I propose estimating *P*_0,*hj*_(*s*, *t*) and $P_{0,hj}^{\prime }(s,t)$, for j∈S and *t* ∈ [*s*, *τ*], via the proposed estimators but using only individuals who were at the transient state *h* at time *s*. More precisely, I propose using the modified counting and at-risk processes ${\tilde{N}}_{im,lj}(t;h,s)=N_{im,lj}(t)Y_{im,h}(s+),\;l\neq j$, and ${\tilde{Y}}_{im,l}(t;h,s)=Y_{im,l}(t)Y_{im,h}(s+),l\in T^{c}$, instead of the original *N*_*im*,*lj*_(*t*) and *Y*_*im*,*l*_(*t*), when estimating *P*_0,*hj*_(*s*, *t*) and $P_{0,hj}^{\prime }(s,t)$, j∈S. These landmark estimators can be shown to be consistent using the same arguments to those used in Putter and Spitoni (2018). Inference with non-Markov processes can be performed as indicated in Theorems 2 and 3, with the exception that the influence functions for the landmark versions of ${\hat{P}}_{n,hj}(s,t)$ and ${\hat{P}}_{n,hj}^{\prime }(s,t)$ involve the modified processes ${\tilde{N}}_{im,lj}(t;h,s)$, *l* ≠ *j*, and ${\tilde{Y}}_{im,l}(t;h,s)$, $l\in T^{c}$. A remark on using Theorems 2 and 3 for inference with non-Markov processes is provided in Web Appendix B.6.

## 3 | SIMULATION STUDIES

**FIGURE 1:**
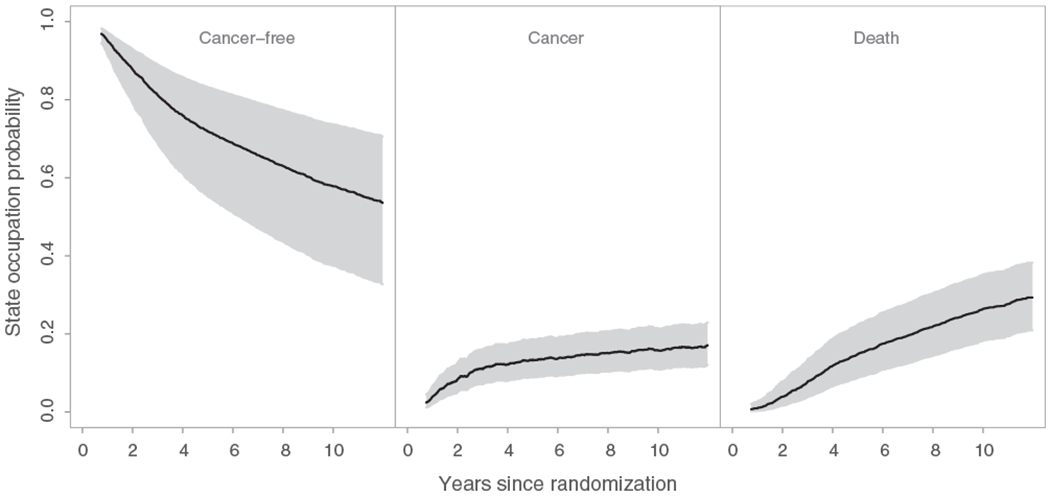
Overall population-averaged state occupation probabilities of the three states (black lines) over the population of all hospital patients in the multicenter EORTC trial 10854, along with the 95% simultaneous confidence bands (gray areas)

To evaluate the small-sample performance of the proposed methods I conducted a series of simulation experiments under a non-Markov illness-death model with states S={1,2,3} and absorbing state T={3}, in a study with ICS. These experiments focused on the population-averaged probabilities *P*_0,2_(*t*), $P_{0,2}^{\prime }(t)$, *P*_0,12_(0.5, *t*), and $P_{0,12}^{\prime }(0.5,t)$. Note that, for the illness-death model where state 1 (healthy) is the unique initial state, *P*_0,2_(*t*) = *P*_0,12_(0, *t*) and $P_{0,2}^{\prime }(t)=P_{0,12}^{\prime }(0,t)$. Scenarios with *n* = 20,40,80 clusters were considered. These sample sizes are considered small or relatively small. The cluster sizes *M_i_*, *i* = 1, … , *n*, were simulated from either of the discrete uniform distributions U(5,15) and U(10,30), producing scenarios with 5 to 15 and 10 to 30 observations per cluster, respectively. To simulate non-Markov illness-death processes, which are correlated within clusters, cluster-specific frailties *v_i_*, *i* = 1 , … , *n*, were simulated from the Gamma distribution with shape and scale parameters equal to 1. Conditionally on the frailty values *v_i_* and the cluster sizes *m_i_*, the non-Markov illness-death processes were simulated based on the cumulative transition intensities *A*_0,12_(*t*; *v_i_*) = [0.25 + 0.25 × *I*{*m_i_* ≤ *E*(*M*_1_)}]*v_i_t*, *A*_0,23_(*t*; *v_i_*) = 0.5*v_i_t*, and *A*_0,13_(*t*; *v_i_*) = 0.25*v_i_t*, *i* = 1, … *n*. Note that the dependence of *A*_0,12_(*t*; *v_i_*) on cluster size produced data with ICS. The resulting population-averaged probabilities of interest are depicted in Figure 1 in Web Appendix D. In this simulation study, two scenarios regarding right censoring and left truncation were considered; the first involved right censoring only while the second considered both right censoring and left truncation. In both scenarios, independent right censoring times were simulated from the uniform distribution *U*(0,3). In the first scenario, the simulation settings led on average to 57.5% right-censored observations (a), 24.4% observations at the illness state (b) (45.9% of those arrived later at the death state), and 18.1% at the death state (c) without a prior visit to the illness state. In the second scenario, left truncation times were independently simulated from the beta distribution Beta(1,2). For the simulations evaluating the estimators of *P*_0,12_(0.5, *t*) and $P_{0,12}^{\prime }(0.5,t)$, this data generation scheme led on average to 67% of the individuals being under observation and at state 1 at time *s* = 0.5. For simulations evaluating state occupation probability estimators, left truncation time was set to 0 with a probability equal to 2/3. This is because estimation of *P*_0,2_(*t*) and $P_{0,2}^{\prime }(t)$ for non-Markov processes under left truncation, involves only individuals who were under observation at time *t* = 0 (see Section 2.6). Therefore, in both cases, around 33% of the observations were excluded from the analysis due to left truncation. Under this setup, a two-arm multicenter randomized controlled trial was also simulated with a 1:1 arm allocation ratio within clusters. To simulate data under the alternative hypothesis, the cumulative intensity *A*_0,*p*12_(*t*; *v_i_*) = [0.25 + 0.5 × *I*(*p* = 2) + 0.25 × *I*{*m_i_* ≤ *E*(*M*_1_)}]*v_i_t*, *p* = 1, 2, was assumed depending on the treatment arm *p*. Estimation of the transition probabilities was performed using the landmark version of the proposed estimators as described in Section 2.6. Simultaneous confidence bands and *P*-values from the Kolmogorov-Smirnov–type tests were based on 1000 simulated sets $\{\xi_{i}{\}}_{i=1}^{n}$ of standard normal variates or 1000 nonparametric cluster bootstrap realizations. Moreover, as described in Section 2.3, the range of the confidence bands was restricted for each data set to the 10th and 90th percentile of the distribution of transition times from state 1 to state 2. We also present simulation results for the one-sample case under the working-independence Aalen-Johansen estimator using the usual Greenwood standard error estimates and a wild bootstrap approach for confidence bands that ignores the within-cluster dependence.

Pointwise simulation results for the state occupation probability estimators under right censoring are presented in Tables 1 and 2. Ignoring the within-cluster dependence was associated with underestimated standard errors and poor coverage probabilities of the 95% confidence intervals. Also, the working independence Aalen-Johansen estimator of $P_{0,2}^{\prime }(t)$ exhibited a small bias as a result of the ICS (relative bias around −7%). The proposed estimators of *P*_0,2_(*t*) and $P_{0,2}^{\prime }(t)$ were both virtually unbiased, the standard error estimates based on the influence functions and the nonparametric cluster bootstrap were both close to the Monte Carlo standard deviation (MCSD) of the estimates, and the corresponding 95% pointwise confidence intervals were close to the nominal level, except for the case with a very small number of clusters (*n*=20) and only 5 to 15 individuals per cluster. It is important to note that the weighted by cluster size working independence estimator ${\hat{P}}_{n,2}^{\prime }(t)$ exhibited a larger MCSD compared to the working independence estimator ${\hat{P}}_{n,2}(t)$ (variance ratio range: 1.15 to 1.21), as a result of the additional variability of the weights.

Simulation results regarding the coverage probabilities of the 95% simultaneous confidence bands are presented in Table 3. The wild bootstrap approach for confidence band calculation that ignores the within-cluster dependence exhibited poor coverage rates in all cases. This phenomenon was more pronounced for the population-averaged state occupation probability $P_{0,2}^{\prime }(\cdot )$ over the TCM population, and is attributed to the bias of the working independence Aalen-Johansen estimator in addition to the variability underestimation. On the contrary, the coverage probabilities of the proposed approaches were close to the nominal level, except for the case with only 20 clusters and 5 to 15 observations per cluster, where the coverage rate was somewhat lower. Finally, simulation results about the empirical rejection rates of the proposed tests are presented in Table 4. Under *H*_0_, the type I error rate of the tests was close to the nominal level *α* = 0.05 in all cases. Under *H*_1_, the empirical power was increasing with sample size and this provides numerical evidence for the consistency of the proposed tests.

Simulation results regarding the estimators of the population-averaged transition probabilities *P*_0,12_(0.5, *t*) and $P_{0,12}^{\prime }(0.5,t)$ under right censoring are presented in Web Appendix D.1. Results under both right censoring and left truncation are presented in Web Appendix D.2. Finally, simulation experiments evaluating the proposed methods under a larger cluster size variability (cluster size range: 5 to 200) and a very small number of clusters (*n* = 15 and 20), are presented in Web Appendix D.3. In all cases, the naïve methods performed poorly. However, this poor performance was less pronounced under both right censoring and left truncation as a result of the fact that, in this case, there were fewer observations per cluster, which led to a less pronounced intracluster dependence issue. The performance of the proposed methods was satisfactory in all cases, with the exception of somewhat lower coverage probabilities (reaching 91% in a few cases) with a very small number of clusters.

## 4 | DATA EXAMPLE

**FIGURE 2:**
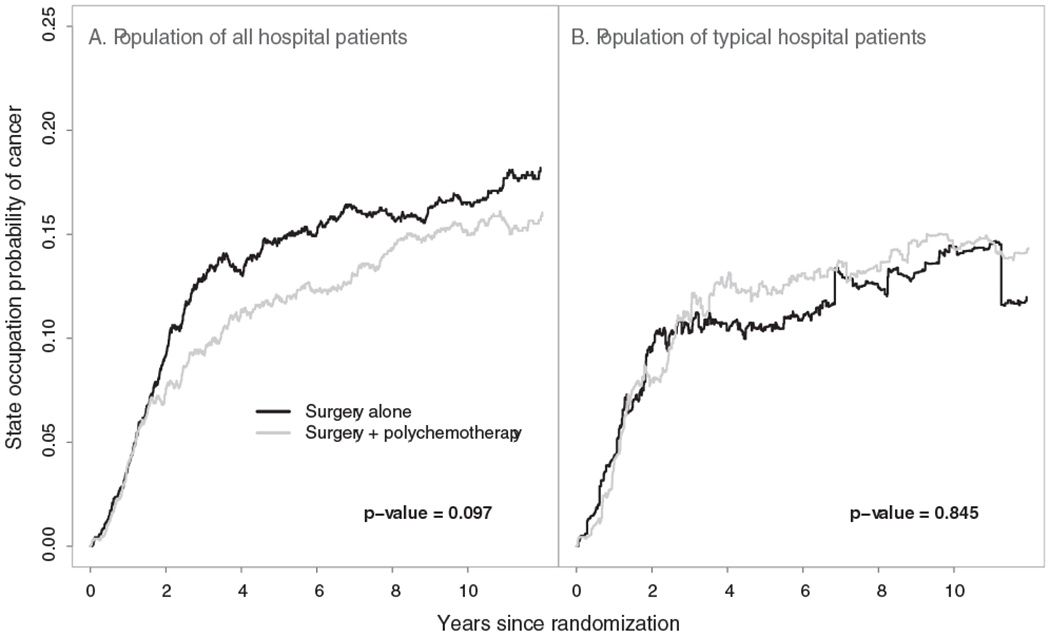
Population-averaged state occupation probabilities of cancer (locoregional relapse, distant metastasis, or secondary cancer) over the population of all hospital patients (A) and the population of typical hospital patients (B) for the two arms in the multicenter EORTC trial 10854, along with the *P*-values from the Kolmogorov-Smirnov–type test

The proposed methods are illustrated using data from the EORTC trial 10854 (Van der Hage et al., 2001). In total, 2792 early breast cancer patients from 15 hospitals (clusters) were recruited in this trial. Of them, 1398 (50.1%) were randomly assigned to the group receiving the combination therapy approach. In this multicenter trial, cluster sizes ranged from 6 to 902 patients. The trial involved only *n* = 15 clusters and thus the analysis based on the proposed large sample inference methods may provide biased results. In this analysis we assumed that the data from the different hospitals are i.i.d. However, the number of patients in one hospital might be correlated with the number of patients in another hospital. This could lead to biased variance estimation and incorrect *P*-values. After surgery, 1146 (41.0%) patients experienced locoregional relapse, distant metastasis, or secondary cancer, and 810 (29.0%) died throughout the follow-up period. Among the deceased patients, 710 (87.7%) died after having experienced a locoregional relapse, distant metastasis, or secondary cancer, while the remaining 100 deceased patients died without prior evidence for these events. The patient event history in this trial can be described by an illness-death model with the states “cancer-free” (state 1), “cancer” (state 2), and “death” (state 3). Throughout the follow-up period, 1546 (55.4%) patients were right-censored while being in the “cancer-free” state and 436 (15.6%) were right-censored while being in the “cancer” state. There was no left truncation in this data set. In this analysis, the focus was on the between-arm comparison of the population-averaged state occupation probabilities of cancer *P*_0,12_(*t*) and $P_{0,12}^{\prime }(t)$ (for the population undergoing surgery only), and *P*_0,22_(*t*) and $P_{0,22}^{\prime }(t)$ (for the population receiving the combination of surgery plus polychemotherapy). The overall state occupation probability estimates for the three states over the population of all hospital patients along with the associated 95% simultaneous confidence bands are presented in Figure 1. These confidence bands were calculated based on 1000 nonparametric cluster bootstrap realizations. Figure 1 provides significant information about the natural history of early breast cancer patients undergoing surgery. The corresponding probabilities for the population of typical hospital patients were approximately the same, with the exception that the probability of cancer was slightly lower in this case (data not shown). The arm-specific state occupation probabilities of cancer for both population of all hospital patients and population of typical hospital patients are presented in Figure 2. To compare these population-averaged probabilities between arms, the proposed Kolmogorov-Smirnov–type test was used based on 1000 nonparametric cluster bootstrap realizations. The tests for both versions of population-averaged probabilities were not statistically significant at the level *α* = 0.05 and, therefore, the null hypothesis that the population-averaged probabilities of cancer do not differ between arms cannot be rejected. Among those in the surgery only group, the estimated population-averaged probability of cancer over the population of typical hospital patients was lower compared to that for the population of all hospital patients (Figure 2). This indicates that larger hospitals had more cancer events among patients with surgery only, which may be attributed to the fact that patients with more advanced disease choose (or are advised to attend) larger hospitals. To evaluate this difference, the modified Kolmogorov-Smirnov–type test described in Web Appendix C was used. The result of this test was statistically significant (*P*-value = .046), which provides evidence for ICS in this group. The corresponding test for the group of patients receiving the combination therapy approach was not statistically significant (*P*-value = .416).

## 5 | DISCUSSION

This work addressed the issue of nonparametric population-averaged inference for multistate models with right-censored and/or left-truncated clustered observations. The estimators for the transition and state occupation probabilities were shown to be uniformly consistent and asymptotically normal with explicit formulas for the corresponding covariance functions. Additionally, rigorous methodology for the calculation of simultaneous confidence bands and a class of Kolmogorov-Smirnov–type tests were proposed. Inference can be performed using either the explicit formulas for the influence functions of the estimators or the nonparametric cluster bootstrap. The latter is particularly useful in practice since it can be used for inference using off-the-shelf software. In this work, I did not impose restrictive distributional assumptions or assumptions regarding the within-cluster dependence. Moreover, I allowed for ICS and nonhomogeneous multistate processes which are non-Markov. Simulation results indicated that the performance of the proposed methods is satisfactory even for non-Markov processes and under ICS. On the contrary, ignoring the within-cluster dependence leads to invalid inference.

The proposed nonparametric estimators of the transition probability matrix and the influence function-based methodology for the calculation of simultaneous confidence bands are extensions of the Aalen-Johansen estimator (Aalen and Johansen, 1978; Andersen et al., 2012) and the wild bootstrap approach for independent data by Bluhmki et al. (2018) to the cluster-correlated data setting. However, these extensions were not trivial given that I allowed for random and ICS. Moreover, I established the asymptotic properties of the proposed methods using empirical process theory instead of martingale theory that was used for the aforementioned methods for independent data (Andersen et al., 2012; Bluhmki et al., 2018). I also considered the nonparametric cluster bootstrap by Field and Welsh (2007). These authors dealt with the case of a simple linear random-intercept model. Even though I used the cluster bootstrap algorithm of Field and Welsh (2007) for the one-sample problem, I proved its consistency for the more complicated nonparametric estimators in Theorem 2. Moreover, for the two-sample problem, the nonparametric cluster bootstrap approach proposed here is slightly different because the weight ${\hat{W}}_{hj}(t)$ is being kept fixed (at its estimated value based on the original data set) across the bootstrap samples, since its variability does not affect the asymptotic null distributions of the test statistics.

It has to be noted that the proposed methods provide large sample inference, as do the typical methods for multistate models. Large sample in the clustered data setting means large number of clusters. Following general recommendations for the central limit theorem, it is suggested to use the proposed methods with at least 30 clusters. However, the extensive simulation studies presented in this article provide some numerical evidence for the satisfactory performance of the proposed methods, and their superiority over the naïve methods that ignore the within-cluster dependence, even with 20 clusters.

I can see two useful extensions of the proposed framework. First, developing an estimation approach for semiparametric regression on the state occupation probabilities would be crucial in practice for the estimation of risk factor effects. This could be achieved by extending the inverse probability of censoring weighting approach by Scheike and Zhang (2007) to the clustered data setting. Second, relaxing the i.i.d. assumption across clusters imposed in this article is important from both theoretical and applied perspective. One situation where this assumption is violated is when there is a dependence between cluster sizes or counting processes from different clusters. A way to deal with this issue is to introduce weak dependence (such as mixing conditions) or long-range dependence assumptions over space or time for the clusters, and use appropriate central limit theorems for such dependent data (Dehling et al., 2002) to establish the asymptotic distributions of the estimators.

## Supplementary Material

Web Appendix

## Figures and Tables

**TABLE 1:** Simulation results for the analysis of *P*_0,2_(*τ*_0.4_) and $P_{0,2}^{\prime }(\tau_{0.4})$, where *τ*_0.4_ is the 40th percentile of the follow-up time, based on the standard approach which ignores the within-cluster dependence (naïve) and the proposed method with (i) the influence function-based variance estimator (IF) and (ii) the nonparametric cluster bootstrap (CB)

			*P*_0,2_(*τ*_0.4_)				$P_{0,2}^{\prime }(\tau_{0.4})$			
				
*n*	*F_M_*	Method	Bias^a^	MCSD^a^	ASE^a^	CP	Bias^a^	MCSD^a^	ASE^a^	CP
20	U[5,15]	Naïve	0.006	3.229	2.625	0.890	−1.022	3.226	2.623	0.859

		IF	0.006	3.229	3.018	0.927	−0.063	3.517	3.311	0.926

		CB	0.006	3.229	3.040	0.928	−0.063	3.517	3.316	0.923

	U[10,30]	Naïve	0.069	2.559	1.857	0.842	−0.928	2.558	1.855	0.816

		IF	0.069	2.559	2.483	0.939	0.077	2.787	2.702	0.940

		CB	0.069	2.559	2.494	0.935	0.077	2.787	2.698	0.939

40	U[5,15]	Naïve	0.105	2.204	1.866	0.909	−0.939	2.199	1.863	0.866

		IF	0.105	2.204	2.196	0.944	0.080	2.403	2.411	0.948

		CB	0.105	2.204	2.198	0.943	0.080	2.403	2.407	0.947

	U[10,30]	Naïve	0.006	1.811	1.312	0.846	−1.003	1.808	1.310	0.779

		IF	0.006	1.811	1.782	0.945	−0.012	1.940	1.941	0.946

		CB	0.006	1.811	1.786	0.944	−0.012	1.940	1.940	0.945

80	U[5,15]	Naïve	−0.037	1.557	1.314	0.903	−1.083	1.551	1.312	0.820

		IF	−0.037	1.557	1.557	0.942	−0.055	1.699	1.715	0.940

		CB	−0.037	1.557	1.556	0.942	−0.055	1.699	1.711	0.940

	U[10,30]	Naïve	0.044	1.287	0.929	0.844	−0.962	1.286	0.928	0.732

		IF	0.044	1.287	1.271	0.945	0.025	1.399	1.382	0.944

		CB	0.044	1.287	1.273	0.944	0.025	1.399	1.382	0.946

Abbreviations: ASE, average estimated standard error; CP, coverage probability; *F_M_*, discrete uniform distribution of the cluster size; MCSD, Monte Carlo standard deviation of the estimates; *n*, number of clusters.

*Note*. Results under right censoring.

a Indicates ×10^2^.

**TABLE 2:** Simulation results for the analysis of *P*_0,2_(τ_0.6_) and $P_{0,2}^{\prime }(\tau_{0.6})$, where *τ*_0.6_ is the 60th percentile of the follow-up time, based on the standard approach that ignores the within-cluster dependence (naïve) and the proposed method with (i) the influence function–based variance estimator (IF) and (ii) the nonparametric cluster bootstrap (CB)

			*P*_0,2_(*τ*_0.6_)				$P_{0,2}^{\prime }(\tau_{0.6})$			
				
*n*	*F_M_*	Method	Bias^a^	MCSD^a^	ASE^a^	CP	Bias^a^	MCSD^a^	ASE^a^	CP
20	U[5,15]	Naïve	0.160	3.657	3.040	0.904	−0.939	3.656	3.033	0.888

		IF	0.160	3.657	3.348	0.921	0.077	3.963	3.651	0.924

		CB	0.160	3.657	3.378	0.916	0.077	3.963	3.663	0.920

	U[10,30]	Naïve	0.116	2.731	2.146	0.869	−0.940	2.740	2.140	0.854

		IF	0.116	2.731	2.679	0.935	0.078	2.978	2.899	0.935

		CB	0.116	2.731	2.693	0.940	0.078	2.978	2.899	0.933

40	U[5,15]	Naïve	0.015	2.360	2.143	0.935	−1.060	2.364	2.140	0.896

		IF	0.015	2.360	2.399	0.955	0.027	2.592	2.635	0.953

		CB	0.015	2.360	2.407	0.957	0.027	2.592	2.636	0.953

	U[10,30]	Naïve	0.035	1.956	1.513	0.866	−1.020	1.943	1.509	0.818

		IF	0.035	1.956	1.915	0.936	−0.011	2.100	2.075	0.937

		CB	0.035	1.956	1.919	0.941	−0.011	2.100	2.075	0.936

80	U[5,15]	Naïve	−0.063	1.745	1.513	0.913	−1.152	1.738	1.510	0.845

		IF	−0.063	1.745	1.714	0.943	−0.084	1.894	1.885	0.949

		CB	−0.063	1.745	1.716	0.945	−0.084	1.894	1.885	0.948

	U[10,30]	Naïve	0.076	1.436	1.073	0.856	−0.972	1.433	1.070	0.775

		IF	0.076	1.436	1.369	0.939	0.045	1.543	1.487	0.942

		CB	0.076	1.436	1.372	0.940	0.045	1.543	1.488	0.945

Abbreviations: ASE, average estimated standard error; CP, coverage probability; *n*, number of clusters; *F_M_*, discrete uniform distribution of the cluster size; MCSD: Monte Carlo standard deviation of the estimates.

*Note*. Results under right censoring.

a Indicates ×10^2^.

**TABLE 3:** Simulation results regarding the coverage probabilities of the 95% simultaneous confidence bands for *P*_0,2_(⋅) and $P_{0,2}^{\prime }(\cdot )$ based on the standard method that ignores the within-cluster dependence (naïve) and the proposed method with (i) the estimated processes ${\hat{B}}_{n,2}$ and ${\hat{B}}_{n,2}^{\prime }$ (IF) and (ii) the nonparametric cluster bootstrap (CB)

		*P*_0,2_(⋅)			$P_{0,2}^{\prime }(\cdot )$		
			
*n*	*F_M_*	Naïve	IF	CB	Naïve	IF	CB
20	U[5,15]	0.856	0.922	0.930	0.826	0.917	0.911

	U[10,30]	0.798	0.944	0.952	0.771	0.946	0.938

40	U[5,15]	0.892	0.948	0.951	0.849	0.945	0.940

	U[10,30]	0.802	0.941	0.942	0.750	0.945	0.946

80	U[5,15]	0.878	0.945	0.943	0.788	0.940	0.942

	U[10,30]	0.820	0.941	0.944	0.689	0.945	0.940

Abbreviations: *F_M_*: discrete uniform distribution of the cluster size; *n*, number of clusters.

*Note*. Results under right censoring.

**TABLE 4:** Simulation results regarding the empirical type I error (*H*_0_) and the empirical power (*H*_1_) of the proposed two-sample Kolmogorov-Smirnov–type tests for *H*_0_ : *P*_0,12_(⋅) = *P*_0,22_(⋅) and $H_{0}\;:P_{0,12}^{\prime }(\cdot )=P_{0,22}^{\prime }(\cdot )$ at the *α* = 0.05 level

		*P*_0,*p*2_(⋅), *p* = 1, 2				$P_{0,p2}^{\prime }(\cdot ),\;p=1,2$			
			
		*H*_0_		*H*_1_		*H*_0_		*H*_1_	
*n*	*F_M_*	IF	CB	IF	CB	IF	CB	IF	CB
20	U[5,15]	0.045	0.042	0.352	0.339	0.049	0.050	0.331	0.337

	U[10,30]	0.040	0.039	0.634	0.625	0.044	0.040	0.598	0.601

40	U[5,15]	0.044	0.041	0.666	0.659	0.037	0.039	0.612	0.603

	U[10,30]	0.048	0.046	0.905	0.906	0.044	0.046	0.874	0.873

80	U[5,15]	0.048	0.046	0.916	0.917	0.049	0.047	0.870	0.864

	U[10,30]	0.053	0.053	0.995	0.994	0.059	0.055	0.991	0.990

Abbreviations: *F_M_*, distribution of the cluster size; *n*: number of clusters.

*Note*. Significance levels were calculated based on either the estimated processes ${\hat{C}}_{n,2}$ and ${\hat{C}}_{n,2}^{\prime }$ (IF) or the nonparametric cluster bootstrap (CB). Results under right censoring.
