# Supplementary material for: Nonparametric analysis of nonhomogeneous multistate processes with clustered observations
Source: Biometrics. 2020 Jul 21;77(2):533–46. doi: 10.1111/biom.13327 (PMC7790918; doi:10.1111/biom.13327)
Supplement: Supplementary file 1 — Web Appendices A, B, C, and D, referenced in Sections 2, 3, and 4, are available with this paper at the Biometrics website on Wiley Online Library. R code for the illness‐death model without recovery, example data, and a README file are also available there. R code for more general processes is available at https://github.com/gbakoyannis/clustered‐multistate. [file BIOM-77-533-s002.pdf]

BIOMETRICS 59, 1–?? DOI: 10.1111/j.1541-0420.2005.00454.x
January 2016

Supporting Information for "Nonparametric Analysis of Nonhomogeneous Multi-State Processes Based on Clustered Observations" by

Giorgos Bakoyannis

Department of Biostatistics, Indiana University, IN 46202, USA

**email:* gbakogia@iu.edu

This paper has been submitted for consideration for publication in *Biometrics*

Web Appendix A: Influence functions

This Web Appendix presents the explicit formulas for the influence functions of the estimators developed in this article. The influence function for the working independence Aalen–Johansen estimator $\hat{P}_{n,hj}(s,t)$, for $h \in \mathcal{T}^c$ and $j \in \mathcal{S}$, with $h \neq j$, is

$$\gamma_{ihj}(s,t) = \sum_{l \in \mathcal{T}^c} \sum_{q \in \mathcal{S}} \int_s^t \frac{P_{0,hl}(s,u-)P_{0,qj}(u,t)}{E\left\{Y_{1\cdot,l}(u)\right\}} d\bar{M}_{ilq}(u), \qquad 0 \leqslant s \leqslant t \leqslant \tau,$$

where

$$\bar{M}_{ilq}(t) = N_{i\cdot,lq}(t) - \int_{(0,t]} Y_{i\cdot,l}(u) dA_{0,lq}(u).$$

If $h = j$, then $\gamma_{ihh}(s,t) = -\sum_{j \neq h} \gamma_{ihj}(s,t)$. The empirical version $\hat{\gamma}_{ihj}(s,t)$ of this influence function can be obtained by replacing the unknown transition probabilities and cumulative transition intensities with their uniformly consistent estimates, and the expectations with sample averages over clusters.

The influence function for the weighted working independence Aalen–Johansen estimator $\hat{P}'_{n,hj}(s,t)$, for $h \in \mathcal{T}^c$ and $j \in \mathcal{S}$, with $h \neq j$, is

$$\gamma'_{ihj}(s,t) = \sum_{l \in \mathcal{T}^c} \sum_{q \in \mathcal{S}} \int_s^t \frac{P'_{0,hl}(s,u-)P'_{0,qj}(u,t)}{E\left\{M_1^{-1} Y_{1\cdot,l}(u)\right\}} d\bar{M}'_{ilq}(u), \qquad 0 \leqslant s \leqslant t \leqslant \tau,$$

where

$$\bar{M}'_{ilq}(t) = M_i^{-1} \left\{ N_{i\cdot,lq}(t) - \int_{(0,t]} Y_{i\cdot,l}(u) dA'_{0,lq}(u) \right\}.$$

If $h = j$, then $\gamma'_{ihh}(s,t) = -\sum_{j \neq h} \gamma'_{ihj}(s,t)$. The empirical version $\hat{\gamma}'_{ihj}(s,t)$ of this influence function can be obtained by replacing the unknown transition probabilities and cumulative transition intensities with their uniformly consistent estimates, and the expectations with sample averages over clusters.

The influence function for the state occupation probability estimator $\hat{P}_{n,j}(t)$ is

$$\begin{aligned}\psi_{ij}(t) \;\; = \;\; & \sum_{h \in \mathcal{T}^c} \Bigg(P_{0,h}(0)\gamma_{ihj}(0,t) + P_{0,hj}(0,t) \Bigg[\frac{Y_{i\cdot,h}(0+) - EY_{1\cdot,h}(0+)}{\pi_0 E M_1} \\ & - P_{0,h}(0) \Bigg\{ \frac{M_i - EM_1}{EM_1} + \frac{M_i^{-1} Y_{i\cdot,\cdot}(0+) - \pi_0}{\pi_0} \Bigg\} \Bigg] \Bigg),\end{aligned}$$

with $Y_{i\cdot,\cdot}(0+) = \sum_{h\in\mathcal{T}^c} Y_{i\cdot,h}(0+)$. The corresponding empirical version can be easily obtained by replacing the unknown state occupation and transition probabilities with their consistent estimates, $\gamma_{ihj}(0,t)$ with $\hat{\gamma}_{ihj}(0,t)$ as described above, and the expectations with sample averages over clusters.

The influence function for the weighted state occupation probability estimator $\hat{P}'_{n,j}(t)$ is

$$\begin{aligned}\psi'_{ij}(t) \;=\; \sum_{h\in\mathcal{T}^c} & (P'_{0,h}(0)\gamma'_{ihj}(0,t) + P'_{0,hj}(0,t)\pi_0^{-1}[M_i^{-1}Y_{i\cdot,h}(0+) - E\{M_1^{-1}Y_{1\cdot,h}(0+)\} \\ & -P'_{0,h}(0)\{M_1^{-1}Y_{1\cdot,\cdot}(0+) - \pi_0\}]).\end{aligned}$$

The corresponding empirical version can be easily obtained by replacing the unknown state occupation and transition probabilities with their consistent estimates, $\gamma'_{ihj}(0,t)$ with $\hat{\gamma}'_{ihj}(0,t)$ as described above, and the expectations with sample averages over clusters.

The classes of functions $\{\psi_{ij}(t) : t \in [0,\tau]\}$ and $\{\psi'_{ij}(t) : t \in [0,\tau]\}$ are P-Donsker for any $j \in \mathcal{S}$. This is due to the fact that these classes consist of linear combinations of functions that belong to P-Donsker classes by Theorem 2 in the main text, fixed functions, and random variables with bounded second moments.

Web Appendix B: Asymptotic Theory Proofs

The proofs of the theorems provided in Section 2 of the manuscript rely on empirical process theory (van der Vaart and Wellner, 1996; Kosorok, 2008). In this Appendix we use the standard empirical processes notation

$$\mathbb{P}_n f = \frac{1}{n}\sum_{i=1}^{n} f(\mathbf{D}_i), \text{ and } Pf = \int_{\mathcal{D}} f dP = Ef,$$

where, for any measurable function $f : \mathcal{D} \mapsto \mathbb{R}$, $\mathbf{D}_i$ denotes the observed variables for the ith cluster, $\mathcal{D}$ denotes the sample space, and P the true (induced) probability measure defined on the Borel σ-algebra on $\mathcal{D}$. We also use the supremum norm notation $\|f(t)\|_\infty \equiv \sup_{t\in[0,\tau]} |f(t)|$. Let V be a generic constant that may differ from place to place. In this Web Appendix we only prove the asymptotic properties of $\hat{\mathbf{P}}_n(s,t)$ since the properties of $\hat{\mathbf{P}}'_n(s,t)$,

$\hat{P}_{n,j}(t)$, and $\hat{P}'_{n,j}(t)$, $j \in \mathcal{S}$, can be established using the same arguments. Without loss of generality and for simplicity of presentation we set the starting point $s = 0$. Before outlining the proofs of Theorems 1-3 we provide and prove two useful lemmas.

Lemma 1: *Let $N(t)$ be an arbitrary counting process on $[0, \tau]$ with $P\{N(\tau)\}^2 < \infty$ and $h(t)$ be a fixed and non-negative function with $h(t) \leqslant V$ almost everywhere with respect to the Lebesgue-Stieltjes measure generated by (the sample paths of) $N(t)$. Then, the class of functions*

$$\mathcal{F}_1(s) = \left\{ \int_s^t h(u)dN(u) : t \in [s, \tau] \right\},$$

is P-Donsker for any $s \in [0, \tau)$.

Proof. Let $\|h\|_{Q,2} = (\int |h|^2 dQ)^{1/2}$ for any probability measure Q. Now, for any probability measure Q and any $t_1, t_2 \in [0, \tau]$ it follows that

$$\begin{aligned} \left\| \int_s^{t_1} h(u)dN(u) - \int_s^{t_2} h(u)dN(u) \right\|_{Q,2} &\leqslant \left\| \int_{t_1}^{t_2} h(u)dN(u) \right\|_{Q,2} \\ &\leqslant V\|N(t_2) - N(t_1)\|_{Q,2}. \end{aligned}$$

By lemma 22.4 in Kosorok (2008), it follows that the class $\Phi_1 = \{N(t) : t \in [0, \tau]\}$ has a bounded uniform entropy integral (BUEI) with envelope $2N(\tau)$, and is also pointwise measurable (PM). This implies that, for any $t \in [0, \tau]$ there exist a $t_i \in [0, \tau]$, $i = 1, \ldots, N(\epsilon 2\|N(\tau)\|_{Q,2}, \Phi_1, L_2(Q))$, such that $\|N(t) - N(t_i)\|_{Q,2} < \epsilon 2\|N(\tau_2)\|_{Q,2}$, for any $\epsilon > 0$ and any finitely discrete probability measure Q. Therefore, for any member of $\mathcal{F}_1(s)$, there exist a $\int_s^{t_i} h(u)dN(u)$, for $i = 1, \ldots, N(\epsilon 2\|N(\tau)\|_{Q,2}, \Phi_1, L_2(Q))$, such that

$$\left\| \int_s^{t} h(u)dN(u) - \int_s^{t_i} h(u)dN(u) \right\|_{Q,2} \leqslant \epsilon 2V\|N(\tau)\|_{Q,2},$$

for any $\epsilon > 0$ and any finitely discrete probability measure Q. Consequently, by the minimality of the covering number it follows that for any $\epsilon > 0$ and any finitely discrete probability measure Q, we have that

$$N(\epsilon 2V\|N(\tau)\|_{Q,2}, \mathcal{F}_1(s), L_2(Q)) \leqslant N(\epsilon 2\|N(\tau)\|_{Q,2}, \Phi_1, L_2(Q)),$$

which yields a BUEI for $\mathcal{F}_1(s)$ with envelope $2VN(\tau)$. Using similar arguments to those used in the example of page 142 of Kosorok (2008), it can be shown that the class $\mathcal{F}_1(s)$ is also PM. Therefore, by Theorem 2.5.2 in van der Vaart and Wellner (1996), the class $\mathcal{F}_1(s)$ is P-Donsker. Since s was arbitrary, the last statement is true for any $s \in [0, \tau)$.

LEMMA 2: *Let $Y(t)$ be an arbitrary at-risk process, $A(t)$ a continuous cumulative transition intensity function on $[0, \tau]$, and $h(t)$ a fixed and non-negative function with $h(t) \leqslant V$ almost everywhere with respect to the Lebesgue-Stieltjes measure generated by $A(t)$. Then, the class of functions*

$$\mathcal{F}_2(s) = \left\{ \int_s^t h(u)Y(u)dA(u) : t \in [0, \tau] \right\}$$

is P-Donsker for any $s \in [0, \tau)$.

Proof. It is not hard to show that for any probability measure Q and any $t_1, t_2 \in [0, \tau]$

$$\left\| \int_s^{t_1} h(u)Y(u)dA(u) - \int_s^{t_2} h(u)Y(u)dA(u) \right\|_{Q,2} \leqslant V|A(t_2) - A(t_1)|.$$

Now, the class of fixed functions $\Phi_2 = \{A(t) : t \in [0, \tau]\}$ is a compact subset of $\mathbb{R}$ as it consists of continuous functions on the compact set $[0, \tau]$. Therefore, this class of fixed functions can be covered by $V(1/\epsilon)$ ϵ-balls and, thus, $N(\epsilon, \Phi_2, |\cdot|) \leqslant V(1/\epsilon)$. Consequently, for any $t \in [0, \tau]$ there exist a $t_i \in [0, \tau]$, $i = 1, \ldots, N(\epsilon, \Phi_2, |\cdot|)$, such that $|A(t) - A(t_i)| < \epsilon$, for any $\epsilon > 0$ and any finitely discrete probability measure Q. Therefore, for any member of $\mathcal{F}_2(s)$, there exist a $\int_s^{t_i} h(u)Y(u)dA(u)$, for $i = 1, \ldots, N(\epsilon, \Phi_2, |\cdot|)$, such that

$$\left\| \int_s^{t} h(u)Y(u)dA(u) - \int_s^{t_i} h(u)Y(u)dA(u) \right\|_{Q,2} \leqslant V\epsilon.$$

for any $\epsilon > 0$ and any finitely discrete probability measure Q. Consequently, by the minimality of the covering number, it follows that for any $\epsilon > 0$ and any finitely discrete probability measure Q, we have that

$$N(\epsilon V, \mathcal{F}_2(s), L_2(Q)) \leqslant V\left(\frac{1}{\epsilon}\right),$$

which yields a BUEI for $\mathcal{F}_2(s)$. Finally, similar arguments to those used in the proof of Lemma 1 lead to the conclusion that the class $\mathcal{F}_2(s)$ is P-Donsker for any $s \in [0, \tau)$.

B.1 *Regularity Conditions*

In this work we assume the following conditions:

C1. The potential left truncation $L_{im,1}$ and right censoring $L_{im,2}$ times are independent of the underlying counting processes $\{\check{N}_{im,hj}(t) : h \neq j, t \in [0, \tau]\}$, the initial state indicators $\check{Y}_{im,h}(0+)$, $h \in \mathcal{T}^c$, and the cluster size M_i. Also, $L_{im,1}$ and $L_{im,2}$ are identically distributed in the sense that $E[\{I(L_{im,1} = 0) + I(L_{im,1} < t)\}I(L_{im,2} \geqslant t)] \equiv ER_{im}(t) = ER_{i1}(t)$, $t \in [0, \tau]$, for any $i = 1, \ldots, n$ and $m = 1, \ldots, M_i$.

C2. The cluster size is bounded in the sense that there exists a (fixed) positive integer m_0 such that $\Pr(M > m_0) = 0$.

C3. The underlying counting processes are identically distributed conditionally on cluster size, which implies that $E\{\check{N}_{im,hj}(t)|M_i\} = E\{\check{N}_{i1,hj}(t)|M_i\}$ for any $i = 1, \ldots, n$, $m = 1, \ldots, M_i$ and $h \neq j$. Also, $E\{\check{N}_{im,hj}(\tau)\}^2 < \infty$ for all $h \neq j$.

C4. The underlying at-risk processes are identically distributed conditionally on cluster size, which implies that $E\{\check{Y}_{im,h}(t)|M_i\} = E\{\check{Y}_{i1,h}(t)|M_i\}$ for any $i = 1, \ldots, n$, $m = 1, \ldots, M_i$ and $h \in \mathcal{S}$. Also, there exists a convex and compact set $J_h \subset [0, \tau]$ such that $\inf_{t \in J_h} E\{\sum_{m=1}^{M_i} \check{Y}_{im,h}(t)\} > 0$ for all $h \in \mathcal{T}^c$, and $\int_{(0,t] \cap J_h^c} dA_{0,hj}(t) = 0$ for all $h \in \mathcal{T}^c$ and $j \neq h$.

C5. The cumulative transition intensities $\{A_{0,hj}(t) : h \neq j, t \in [0, \tau]\}$ are continuous functions.

C6. Strengthen condition C4 to require $\inf_{t \in [0,\tau]} E\{\sum_{m=1}^{M_i} \check{Y}_{im,h}(t)\} > 0$ for all $h \in \mathcal{T}^c$.

Condition C1 implies that the right censoring and left truncation times are independent of the at-risk processes $\{\check{Y}_{im,h}(t) : h \in \mathcal{T}^c, t \in [0, \tau]\}$. Conditions C1 (except for the independence between cluster size and right censoring/left truncation), C5, and the second parts of conditions C3 and C4 ensure that the standard Aalen–Johansen estimator (Aalen

and Johansen, 1978) of $\mathbf{P}_0$ based on i.i.d. data is uniformly consistent and its elements convergence weakly to tight (and possibly degenerate) Gaussian processes. The additional conditions needed for the situation with clustered data are that cluster sizes are bounded (condition C2), the right censoring and left truncation times are independent of the cluster size, and that the counting and at-risk processes are identically distributed within clusters, conditionally on cluster size. These additional conditions are realistic in practical applications. It has to be mentioned that a violation of the additional condition C6 is associated with weak convergence to degenerate Gaussian processes for the proposed estimators. In Web Appendix A.5 we relax condition C6 and discuss the practical implications of this.

For the nonparametric two-sample Kolmogorov–Smirnov tests we refine conditions C3, C4 and C6 as follows:

C3'. The underlying counting processes are identically distributed conditionally on cluster size, which implies that $E\{\check{N}_{ipm,hj}(t)|M_{pi}\} = E\{\check{N}_{ip1,hj}(t)|M_{pi}\}$ for any $i = 1, \ldots, n$, $p = 1, 2$, $m = 1, \ldots, M_{pi}$ and $h \neq j$. Also, $E\{\check{N}_{ipm,hj}(\tau)\}^2 < \infty$ for all $h \neq j$.

C4'. The underlying at-risk processes are identically distributed conditionally on cluster size, which implies that $E\{\check{Y}_{ipm,h}(t)|M_{pi}\} = E\{\check{Y}_{ip1,h}(t)|M_{pi}\}$ for any $i = 1, \ldots, n$, $p = 1, 2$, $m = 1, \ldots, M_{pi}$ and $h \in \mathcal{S}$. Also, there exists a compact set $J_h \subset [0, \tau]$ such that $\inf_{t \in J_h} E\{\sum_{m=1}^{M_{pi}} \check{Y}_{ipm,h}(t)\} > 0$, $p = 1, 2$, for all $h \in \mathcal{T}^c$, and $\int_{(0,\tau] \cap J_h^c} dA_{0,phj}(t) = 0$, $p = 1, 2$, for all $h \in \mathcal{T}^c$ and $j \neq h$.

C6'. Strengthen condition C4' to require $\inf_{t \in [0,\tau]} E\{\sum_{m=1}^{M_{pi}} \check{Y}_{ipm,h}(t)\} > 0$, $p = 1, 2$, for all $h \in \mathcal{T}^c$.

Note that the counting and at-risk processes are also allowed to depend on the total cluster size M_i. However, the assumption of identical distributions in conditions C3' and C4' is defined conditional on the size of the pth sample within the ith cluster.

B.2 *Proof of Theorem 1*

It is clear that $\check{N}_{im,hj}(t)$, $h \neq j$ can be expressed as

$$\begin{aligned}\check{N}_{im,hj}(t) &= \sum_{v=1}^{\check{N}_{im,hj}(\tau)} I(T_{imv,hj} \leqslant t) \\ &= \sum_{v=1}^{v_0} I(v \leqslant \check{N}_{im,hj}(\tau), T_{imv,hj} \leqslant t), \quad a.s.\end{aligned}$$

where $T_{imv,hj}$, $v = 1, \dots, \check{N}_{im,hj}(\tau)$, are the random jump times of $\check{N}_{im,hj}(t)$, $t \in [0, \tau]$, and $v_0 \in \mathbb{N}$ is a constant which is selected to satisfy $\check{N}_{im,hj}(\tau) \leqslant v_0$ a.s. in light of condition C3. The corresponding observable version, which is subject to right censoring and/or left truncation, is

$$\begin{aligned}N_{im,hj}(t) &= \sum_{v=1}^{\check{N}_{im,hj}(\tau)} I(T_{imv,hj} \leqslant t, R_{im}(T_{imv,hj}) = 1) \\ &= \sum_{v=1}^{v_0} I(v \leqslant \check{N}_{im,hj}(\tau), T_{imv,hj} \leqslant t, R_{im}(T_{imv,hj}) = 1), \quad a.s.\end{aligned}$$

Thus, by conditions C1 and C2,

$$\begin{aligned}EN_{i\cdot,hj}(t) &= \sum_{m=1}^{m_0} \sum_{v=1}^{v_0} \Pr(m \leqslant M_i, v \leqslant \check{N}_{im,hj}(\tau), T_{imv,hj} \leqslant t, R_{im}(T_{imv,hj}) = 1) \\ &= \int_0^t E\{R_{i1}(u)\} dE \sum_{m=1}^{m_0} \sum_{v=1}^{v_0} I(m \leqslant M_i, v \leqslant \check{N}_{im,hj}(\tau), T_{imv,hj} \leqslant u) \\ &= \int_0^t E\{R_{i1}(u)\} dE\check{N}_{i\cdot,hj}(u), \qquad t \in [0, \tau].\end{aligned}$$

Additionally, the observed version of $\check{Y}_{im,h}(t)$, $h \in \mathcal{T}^c$, is $Y_{im,h}(t) = \check{Y}_{im,h}(t) R_{im}(t-)$, $t \in [0, \tau]$ and thus, by conditions C1, C2, and C5

$$EY_{i\cdot,h}(t) = E\{R_{i1}(t)\} E\{\check{Y}_{i\cdot,h}(t)\}, \qquad t \in [0, \tau].$$

Next, in light of the assumption of identically distributed counting processes conditional on cluster size (condition C3), condition C2, and the i.i.d. assumption of the observations across

clusters it follows that for $h \neq j$ and any $t \in [0, \tau]$

$$\begin{aligned} E\check{N}_{1\cdot,hj}(t) &= E\left[E\left\{\sum_{m=1}^{M_1} \check{N}_{1m,hj}(t)\middle| M_1\right\}\right] \\ &= E\left[E\left\{\check{N}_{11,hj}(t)\middle| M_1\right\}\sum_{m=1}^{m_0} I(m \leqslant M_1)\right] \\ &= E\left[E\left\{\check{N}_{1m,hj}(t)\middle| M_1\right\} M_1\right] \\ &= E\left\{M_1\check{N}_{1m,hj}(t)\right\}, \quad t \in [0,\tau], \end{aligned}$$

for any $m = 1, \ldots, M_1$. Similarly, under conditions C2 and C4, it can be shown that $E\check{Y}_{1\cdot,h}(t) = E\{M_1\check{Y}_{1m,h}(t)\}$, $h \in \mathcal{T}^c$, $t \in [0, \tau]$, for any cluster member $m = 1, \ldots, M_1$. As a result,

$$A_{0,hj}(t) = \int_0^t \frac{dE\{M_1\check{N}_{1m,hj}(u)\}}{E\{M_1\check{Y}_{1m,h}(u)\}} = \int_0^t \frac{dE\check{N}_{1\cdot,hj}(u)}{E\check{Y}_{1\cdot,h}(u)}, \qquad h \neq j$$

Taking all the pieces together, and using empirical process theory notation, if follows, by condition C4, that

$$\begin{aligned} \int_0^t \frac{dPN_{\cdot,hj}(u)}{PY_{\cdot,h}(u)} &= \int_{(0,t]\cap J_h} \frac{dPN_{\cdot,hj}(u)}{PY_{\cdot,h}(u)} \\ &= \int_{(0,t]\cap J_h} \frac{PR_1(u)dP\check{N}_{\cdot,hj}(u)}{PR_1(u)P\check{Y}_{\cdot,h}(u)} \\ &= A_{0,hj}(t), \end{aligned}$$

since condition C4 ensures $\inf_{t\in[0,t]\cap J_h} PR_{\cdot,h}(t) > 0$ and $\int_{(0,t]\cap J_h^c} dA_{0,hj}(t) = 0$. Next, it is easy to see that, for any $h \in \mathcal{T}^c$ and $j \in \mathcal{S}$, the following inequality holds:

$$\begin{aligned} \left\|\hat{A}_{n,hj}(t) - A_{0,hj}(t)\right\|_\infty &\leqslant \left\|\mathbb{P}_n \int_{(0,t]} \left\{\frac{1}{\mathbb{P}_n Y_{\cdot,h}(u)} - \frac{1}{PY_{\cdot,h}(u)}\right\} dN_{\cdot,hj}(u)\right\|_\infty \\ &\quad + \left\|(\mathbb{P}_n - P)\int_{(0,t]} \frac{dN_{\cdot,hj}(u)}{PY_{\cdot,h}(u)}\right\|_\infty \\ &\equiv Q_{n,1} + Q_{n,2}. \qquad (1) \end{aligned}$$

The first term can be bounded as follows:

$$\begin{aligned} Q_{n,1} &\leqslant \|\mathbb{P}_n Y_{\cdot,h}(t) - PY_{\cdot,h}(t)\|_\infty \left\| \mathbb{P}_n \int_{(0,t]} \frac{dN_{\cdot,hj}(u)}{\mathbb{P}_n Y_{\cdot,h}(u) PY_{\cdot,h}(u)} \right\|_\infty \\ &\leqslant V\|\mathbb{P}_n Y_{\cdot,h}(t) - PY_{\cdot,h}(t)\|_\infty \left\| \mathbb{P}_n \int_{(0,t]} \frac{dN_{\cdot,hj}(u)}{\mathbb{P}_n Y_{\cdot,h}(u)} \right\|_\infty \end{aligned}$$

where the last inequality follows from condition C4, which implies that there exists a constant V such that $\{PY_{\cdot,h}(t)\}^{-1} \leqslant V$ a.e. $(\mu_{N_{\cdot,hj}})$, with $\mu_{N_{\cdot,hj}}$ being the Lebesgue–Stieltjes measure generated by (the sample paths of) $N_{\cdot,hj}(t)$. By conditions C2 and C3, the class of functions $\{Y_{\cdot,h}(t) = \sum_{m=1}^{m_0} I(m \leqslant M) Y_{m,h}(t) : t \in [0,\tau]\}$ can be expressed as a (finite) linear combination of monotone caglad square-integrable processes (Andersen et al., 2012), multiplied by $R_m(t-)$, which belong to Donsker classes by lemma 4.1 in Kosorok (2008). Thus, by lemma 4.1 and corollary 9.32 in Kosorok (2008), the classes $\{Y_{\cdot,h}(t) : t \in [0,\tau]\}$, $h \in \mathcal{T}^c$, are P-Donsker and, therefore, also P-Glivenko–Cantelli. Consequently, $\|\mathbb{P}_n Y_{\cdot,h}(t) - PY_{\cdot,h}(t)\|_\infty \overset{as*}{\to} 0$. This result and the fact that $\{\mathbb{P}_n Y_{\cdot,h}(t)\}^{-1}$ is bounded a.e. $(\mu_{N_{\cdot,hj}})$ with probability 1 lead to the conclusion that $Q_{n,1} \overset{as*}{\to} 0$. For $Q_{n,2}$, conditions C1 and C4 imply that there exists a constant V such that

$$\frac{1}{PY_{\cdot,h}(t)} \leqslant V \quad a.e. \;\; (\mu_{N_{\cdot,hj}}).$$

Thus, by conditions C2, C3, and Lemma 1, it follows that the class $\{\int_{(0,t]} \{PY_{\cdot,h}(u)\}^{-1} dN_{\cdot,hj}(u) : t \in [0,\tau]\}$ is P-Donsker and thus also P-Glivenko–Cantelli. This implies that $Q_{n,2} \overset{as*}{\to} 0$ and, consequently, by inequality (1) it follows that $\|\hat{A}_{n,hj}(t) - A_{0,hj}(t)\|_\infty \overset{as*}{\to} 0$, for all $h \in \mathcal{T}^c$ and $j \in \mathcal{S}$. This result along with the continuity of the product integral (Andersen et al., 2012) lead to the conclusion that

$$\prod_{(0,t]} \{\mathbf{I}_k + d\hat{\mathbf{A}}_n(u)\} \overset{as*}{\to} \prod_{(0,t]} \{\mathbf{I}_k + d\mathbf{A}_0(u)\},$$

uniformly in $t \in [0,\tau]$.

B.3 *Proof of Theorem 2*

The class of functions $\{N_{\cdot,hj}(t) = \sum_{m=1}^{m_0} I(m \leqslant M)N_{m,hj}(t) : [0,\tau]\}$ is P-Donsker for any $h \in \mathcal{T}^c$ and $j \in \mathcal{S}$, by conditions C2 and C3, and lemma 4.1 and corollary 9.32 in Kosorok (2008). Also, the class $\{Y_{\cdot,h}(t) : [0,\tau]\}$ is P-Donsker for any $h \in \mathcal{T}^c$ as argued in the proof of Theorem 1. Therefore

$$\sqrt{n}\begin{pmatrix} \mathbb{P}_n N_{\cdot,hj} - PN_{\cdot,hj} \\ \mathbb{P}_n Y_{\cdot,h} - PY_{\cdot,h} \end{pmatrix} \rightsquigarrow \begin{pmatrix} \tilde{\mathbb{G}}_{1hj} \\ \tilde{\mathbb{G}}_{2h} \end{pmatrix} \quad \text{in} \quad (D[0,\tau])^2,$$

for $h \neq j$, where $\tilde{\mathbb{G}}_{1hj}$ and $\tilde{\mathbb{G}}_{2h}$ are tight zero mean Gaussian processes with covariance functions $PN_{\cdot,hj}(t_1)N_{\cdot,hj}(t_2) - PN_{\cdot,hj}(t_1)PN_{\cdot,hj}(t_2)$ and $PY_{\cdot,h}(t_1)Y_{\cdot,h}(t_2) - PY_{\cdot,h}(t_1)PY_{\cdot,h}(t_2)$, respectively, for $t_1, t_2 \in [0,\tau]$. The cross-covariance between $\tilde{\mathbb{G}}_{1hj}(t_1)$ and $\tilde{\mathbb{G}}_{2h}(t_2)$ is $PN_{\cdot,hj}(t_1)Y_{\cdot,h}(t_2) - PN_{\cdot,hj}(t_1)PY_{\cdot,h}(t_2)$. Moreover, the map $(F_1, F_2) \mapsto \int_{[0,t]} F_1^{-1} dF_2$ is Hadamard differentiable on the domain

$$\left\{(F_1, F_2) : \inf_{t\in[0,\tau]} |F_1(t)| \geqslant \epsilon, \int_{[0,\tau]} |dF_2(t)| < \infty\right\}$$

for $\epsilon > 0$ and F_1^{-1} of bounded variation (Kosorok, 2008), with derivative at (f_1, f_2) given by

$$\int_{[0,t]} \frac{df_1}{F_2} - \int_{[0,t]} \frac{f_2}{F_2^2} dF_1.$$

These facts along with condition C6 and the functional delta method (van der Vaart, 2000), lead to the conclusion that

$$
\begin{aligned}
\sqrt{n}\{\hat{A}_{n,hj}(t) - A_{0,hj}(t)\} &= \sqrt{n}\mathbb{P}_n\Bigg[\int_{(0,t]} \frac{d\{(\mathbb{P}_n - P)N_{\cdot,hj}(u)\}}{PY_{\cdot,h}(u)} \\
&\quad - \int_{(0,t]} \frac{(\mathbb{P}_n - P)Y_{\cdot,h}(u)}{PY_{\cdot,h}(u)} dA_{0,hj}(u)\Bigg] + o_p(1) \\
&= \sqrt{n}\mathbb{P}_n\left\{\int_{(0,t]} \frac{dN_{\cdot,hj}(u)}{PY_{\cdot,h}(u)} - \int_{(0,t]} \frac{Y_{\cdot,h}(u)}{PY_{\cdot,h}(u)} dA_{0,hj}(u)\right\} \\
&\quad -\sqrt{n}\left\{\int_{(0,t]} \frac{dPN_{\cdot,hj}(u)}{PY_{\cdot,h}(u)} - A_{0,hj}(t)\right\} + o_p(1) \\
&= \sqrt{n}\mathbb{P}_n\left\{\int_{(0,t]} \frac{dN_{\cdot,hj}(u)}{PY_{\cdot,h}(u)} - \int_{(0,t]} \frac{Y_{\cdot,h}(u)}{PY_{\cdot,h}(u)} dA_{0,hj}(u)\right\} + o_p(1) \\
&= \sqrt{n}\mathbb{P}_n \int_{(0,t]} \frac{d\bar{M}_{hj}(u)}{PY_{\cdot,h}(u)} + o_p(1) \\
&\equiv \sqrt{n}\mathbb{P}_n\phi_{hj}(t) + o_p(1), \quad t \in [0,\tau].
\end{aligned}
$$

The class of the influence functions $\{\phi_{hj}(t) : t \in [0,\tau]\}$ is P-Donsker by the Donsker property of the class $\{N_{\cdot,hj}(t) : [0,\tau]\}$, conditions C2–C5, Lemmas 1 and 2, and corollary 9.32 in Kosorok (2008). Therefore, $\sqrt{n}(\hat{A}_{n,hj} - A_{0,hj})$ converges weakly to a tight zero mean Gaussian process $\tilde{\mathbb{G}}_{3hj}$ in $D[0,\tau]$ with covariance function $P\phi_{hj}(t_1)\phi_{hj}(t_2)$, $t_1, t_2 \in [0,\tau]$, for $h \neq j$. For $h = j$, $\sqrt{n}\{\hat{A}_{n,hh}(t) - A_{0,hh}(t)\} = -\sqrt{n}\mathbb{P}_n \sum_{h \neq j} \phi_{hj}(t) + o_p(1)$, where the influence functions belong obviously to a P-Donsker class. Thus, the joint sequence $\sqrt{n}(\hat{A}_{n,hj} - A_{0,hj})$ for $h \neq j$, converges weakly to a tight zero mean Gaussian process with cross-covariance between $\tilde{\mathbb{G}}_{3hj}(t_1)$ and $\tilde{\mathbb{G}}_{3lq}(t_2)$ equal to $P\phi_{hj}(t_1)\phi_{lq}(t_2)$, for $h \neq j$, $l \neq q$, $t_1, t_2 \in [0,\tau]$. Therefore, $\sqrt{n}(\hat{\mathbf{A}}_n - \mathbf{A}_0)$ converges weakly to a tight zero mean Gaussian process in $(D[0,\tau])^{k^2}$. Now, the Hadamard differentiability of the product integral map (proposition II.8.7 in Andersen et al., 2012)

$$
\mathbf{A}_0 \mapsto \prod(\mathbf{I}_k - d\mathbf{A}_0),
$$

and the functional delta method (van der Vaart, 2000; Andersen et al., 2012) lead to the conclusion that

$$\begin{aligned}\sqrt{n}\{\hat{\mathbf{P}}_n(0,t)-\mathbf{P}_0(0,t)\} &= \sqrt{n}\mathbb{P}_n\int_0^t \prod_{[0,u)}\{\mathbf{I}_k+d\mathbf{A}_0(v)\}\boldsymbol{\phi}(du)\prod_{(u,\cdot]}\{\mathbf{I}_k+d\mathbf{A}_0(v)\}+o_p(1)\\ &\equiv \sqrt{n}\mathbb{P}_n\boldsymbol{\gamma}(0,t)+o_p(1), \qquad t\in[0,\tau]\end{aligned}$$

where the matrix $\boldsymbol{\phi}_i(t)$ contains the elements $\phi_{ihj}(t)$, and the matrix $\boldsymbol{\gamma}_i(0,t)$ contains the elements

$$\gamma_{ihj}(0,t)=\sum_{l\in\mathcal{T}^c}\sum_{q\in\mathcal{S}}\int_0^t \frac{P_{0,hl}(0,u-)P_{0,qj}(u,t)}{PY_{\cdot,l}(u)}d\bar{M}_{ilq}(u), \qquad t\in[0,\tau].$$

By the P-Donsker property of the classes $\{N_{\cdot,hj}(t):t\in[0,\tau]\}$, for $h\neq t$, and $\{Y_{\cdot,h}(t):t\in[0,\tau]\}$, for $h\in\mathcal{T}^c$, conditions C3-C5, corollary 9.32 in Kosorok (2008), and Lemmas 1 and 2, it follows that the classes $\{\gamma_{hj}(0,t):t\in[0,\tau]\}$ are P-Donsker for all $h\in\mathcal{T}^c$, $j\in\mathcal{S}$. This concludes the proof of part (i) of Theorem 2.

Before showing the weak convergence results which are conditional on the observed data, we provide a more formal definition of what does conditional weak convergence mean. Clearly, conditionally on the observed data, the only source of randomness in $\hat{B}_{n,hj}(s,\cdot)$ and $\hat{B}'_{n,hj}(s,\cdot)$ are the standard normal variates ξ_i. Weak convergence of conditional laws of a random sequence $G_n(\xi,\mathbf{O})$, that depends on the simulation realizations ξ and the observed data $\mathbf{O}$, to a tight process G in some metric space $(\mathbb{D},d)$ is defined as follows (Kosorok, 2008)

$$\sup_{f\in BL_1}|E_\xi f[G_n(\xi,\mathbf{O})]-Ef(G)|\overset{p}{\to}0,$$

where BL_1 is the space of Lipschitz functions $f:\mathbb{D}\mapsto\mathbb{R}$, with Lipschitz norm bounded by 1, and E_ξ denotes conditional expectation with respect to the simulation realizations ξ, given the observed data $\mathbf{O}$. This type of weak convergence is denoted as $G_n(\xi,\mathbf{O})\underset{\xi}{\overset{p}{\rightsquigarrow}}G$. Weak convergence of conditional laws of the cluster bootstrap processes is defined in a similar manner. More precisely, let $(U_{n1},\ldots,U_{nn})$ be a random vector from the multinomial distribution with n trials and probabilities $1/n$ for each trial. Then, the nonparametric cluster

bootstrap versions of the proposed estimators are

$$\hat{\mathbf{P}}_n^*(s,t) = \prod_{(s,t]} \{\mathbf{I}_k + d\hat{\mathbf{A}}_n^*(u)\}$$

and

$$\hat{\mathbf{P}}_n'^*(s,t) = \prod_{(s,t]} \{\mathbf{I}_k + d\hat{\mathbf{A}}_n'^*(u)\},$$

where $\hat{\mathbf{A}}_n^*(t)$ and $\hat{\mathbf{A}}_n'^*(t)$ involve the components

$$\hat{A}^*_{n,hj}(t) = \int_0^t \frac{d\left\{\sum_{i=1}^n U_{ni} N_{i\cdot,hj}(u)\right\}}{\sum_{i=1}^n U_{ni} Y_{i\cdot,h}(u)}, \quad h \neq j, \quad t \in [0,\tau],$$

and

$$\hat{A}'^*_{n,hj}(t) = \int_0^t \frac{d\left\{\sum_{i=1}^n U_{ni} M_i^{-1} N_{i\cdot,hj}(u)\right\}}{\sum_{i=1}^n U_{ni} M_i^{-1} Y_{i\cdot,h}(u)}, \quad h \neq j, \quad t \in [0,\tau],$$

respectively. Weak convergence of conditional laws of the corresponding bootstrap processes is defined, conditionally on the observed data, with respect to the multinomial bootstrap weights U and is denoted as $\underset{U}{\overset{p}{\rightsquigarrow}}$.

For the first conditional weak convergence result in part (ii) of Theorem 2, define the process $\tilde{B}_{hj}(0,t) = \sqrt{n}\mathbb{P}_n \gamma_{hj}(0,t)\xi$. By the P-Donsker property of the class $\{\gamma_{hj}(0,t) : t \in [0,\tau]\}$ and the conditional multiplier central limit theorem (Kosorok, 2008) it follows that $\tilde{B}_{hj}(0,\cdot) \underset{\xi}{\overset{p}{\rightsquigarrow}} \mathbb{G}_{hj}(0,\cdot)$. Thus, it remains to show that

$$\|\hat{B}_{hj}(0,t) - \tilde{B}_{hj}(0,t)\|_\infty = o_p(1),$$

unconditionally on the observed data. After some algebra it can be shown that

$$\|\hat{B}_{hj}(0,t) - \tilde{B}_{hj}(0,t)\|_\infty \leqslant \sum_{l\in\mathcal{T}^c} \sum_{q\in\mathcal{S}} (\tilde{Q}_{n,lq1} + \tilde{Q}_{n,lq2} + \tilde{Q}_{n,lq3}), \tag{2}$$

where

$$\tilde{Q}_{n,lq1} = \left\| \sqrt{n}\mathbb{P}_n \int_0^t \left\{ \frac{\hat{P}_{n,hl}(0,u-)\hat{P}_{n,qj}(u,t)}{\mathbb{P}_n Y_{\cdot,l}(u)} - \frac{P_{0,hl}(0,u-)P_{0,qj}(u,t)}{PY_{\cdot,l}(u)} \right\} dN_{\cdot,lq}(u)\xi \right\|_\infty,$$

$$\tilde{Q}_{n,lq2} = \left\| \sqrt{n}\mathbb{P}_n \int_0^t \left\{ \frac{\hat{P}_{n,hl}(0,u-)\hat{P}_{n,qj}(u,t)}{\mathbb{P}_n Y_{\cdot,l}(u)} - \frac{P_{0,hl}(0,u-)P_{0,qj}(u,t)}{PY_{\cdot,l}(u)} \right\} d\hat{A}_{n,lq}(u)\xi \right\|_\infty,$$

and

$$\tilde{Q}_{n,lq3} = \left\| \int_0^t \frac{P_{0,hl}(0,u-)P_{0,qj}(u,t)}{PY_{\cdot,l}(u)} \{\sqrt{n}\mathbb{P}_n Y_{\cdot,l}(u)\xi\} d\{\hat{A}_{n,lq}(u) - A_{0,lq}(u)\} \right\|_\infty .$$

Next, it is easy to see that

$$\left| \frac{\hat{P}_{n,hl}(0,u-)\hat{P}_{n,qj}(u,t)}{\mathbb{P}_n Y_{\cdot,l}(u)} - \frac{P_{0,hl}(0,u-)P_{0,qj}(u,t)}{PY_{\cdot,l}(u)} \right| \leqslant V \Bigg\{ \sup_{u\in[0,t]} |\hat{P}_{n,hl}(0,u-) - P_{0,hl}(0,u-)| + \sup_{u\in[0,t]} |\hat{P}_{n,hl}(u,t) - P_{0,hl}(u,t)| + \sup_{u\in[0,t]} \left| \frac{1}{\mathbb{P}_n Y_{\cdot,l}(u)} - \frac{1}{PY_{\cdot,l}(u)} \right| \Bigg\},$$

almost everywhere with respect to both $\mu_{N_{\cdot,lq}}$ and $\mu_{\hat{A}_{n,lq}}$ (which is the Lebesgue–Stieltjes measure generated by $\hat{A}_{n,lq}$). Therefore, by condition C3 and C6, the outer almost sure consistency of the transition probability estimators, arguments similar to those used in the proof of Theorem 1, and the central limit theorem, it follows that

$$\tilde{Q}_{n,lq1} \leqslant o_{as*}(1)O_p(1)V = o_p(1).$$

By similar arguments and condition C5 it follows that $\tilde{Q}_{n,lq2} = o_p(1)$. Finally, by the P-Donsker property of the class $\{Y_{\cdot,l}(t) : t \in [0,\tau]\}$, the uniform consistency of the cumulative transition intensity, and the same arguments to those used in the proof of proposition 7.27 in Kosorok (2008), it follows that $\tilde{Q}_{n,lq3} = o_p(1)$, since convergence in distribution to a constant implies convergence in probability. Thus, by (2), $\|\hat{B}_{hj}(0,t) - \tilde{B}_{hj}(0,t)\|_\infty = o_p(1)$ and this concludes the proof of the first conditional weak convergence result in part (ii) of Theorem 2.

For the second conditional weak convergence result in part (ii) of Theorem 2, the P-Donsker property of the classes $\{N_{\cdot,hj}(t) : t \in [0,\tau]\}$ and $\{Y_{\cdot,h}(t) : t \in [0,\tau]\}$, condition C3, the weak convergence of the sequence $\sqrt{n}(\hat{A}_{n,hj} - A_{0,hj})$, the bootstrap central limit theorem (Kosorok, 2008), and the bootstrap functional delta method (Kosorok, 2008, Theorem 12.1), imply that $\sqrt{n}(\hat{A}^*_{n,hj} - \hat{A}_{n,hj}) \underset{U}{\overset{p}{\rightsquigarrow}} \mathbb{G}_{3hj}$ in $D[0,\tau]$, for $h \in \mathcal{T}^c$ and $j \neq h$. A second application of the bootstrap functional delta method and the bootstrap continuous mapping theorem (Theorem

10.8, Kosorok, 2008) lead to the conclusion that $\sqrt{n}\{\hat{P}^*_{n,hj}(0,\cdot)-\hat{P}_{n,hj}(0,\cdot)\} \overset{p}{\underset{U}{\rightsquigarrow}} \mathbb{G}_{hj}(0,\cdot)$. The proof of part (iii) of Theorem 2 follows from the same arguments.

B.4 *Proof of Theorem 3*

By Theorem 2 and the uniform consistency of $\hat{W}_{hj}(t)$, it follows that

$$\begin{aligned}\sqrt{n}\hat{W}_{hj}(t)\hat{\Delta}(0,t) &= \{\hat{W}_{hj}(t)-W_{hj}(t)\}\sqrt{n}\mathbb{P}_n\{\gamma_{1,hj}(0,t)-\gamma_{2,hj}(0,t)\}\\ &\quad+\sqrt{n}\mathbb{P}_n W_{hj}(t)\{\gamma_{1,hj}(0,t)-\gamma_{2,hj}(0,t)\}+o_p(1)\\ &= \sqrt{n}\mathbb{P}_n W_{hj}(t)\{\gamma_{1,hj}(0,t)-\gamma_{2,hj}(0,t)\}+o_p(1).\end{aligned}$$

The boundedness of the fixed function $W_{hj}(t)$ and the P-Donsker property of $\{\gamma_{p,hj}(0,t) : t \in [0,\tau]\}$, $p = 1,2$, imply that the class $\{W_{hj}(t)\{\gamma_{1,hj}(0,t)-\gamma_{2,hj}(0,t)\} : t \in [0,\tau]\}$ is P-Donsker. Therefore, $\sqrt{n}\hat{W}_{hj}(\cdot)\hat{\Delta}(0,\cdot) \rightsquigarrow \mathbb{Z}_{hj}(0,\cdot)$ in $D[0,\tau]$, with the covariance function of the process $\mathbb{Z}_{hj}(0,\cdot)$ being

$$W_{hj}(t_1)W_{hj}(t_2)P[\{\gamma_{1,hj}(0,t_1)-\gamma_{2,1hj}(0,t_1)\}\{\gamma_{1,hj}(0,t_2)-\gamma_{2,1hj}(0,t_2)\}],$$

for $t_1, t_2 \in [0,\tau]$.

Next, by the conditional multiplier central limit theorem it follows that

$$\tilde{C}_{n,hj}(0,\cdot) \equiv \sqrt{P}_n W_{hj}(\cdot)\{\gamma_{1,hj}(0,\cdot)-\gamma_{2,hj}(0,\cdot)\}\xi \overset{p}{\underset{\xi}{\rightsquigarrow}} \mathbb{Z}_{hj}(0,\cdot) \quad \textit{in} \;\; D[0,\tau].$$

Also, by the uniform boundedness of $W_{hj}(t)$ and the P-Donsker property of the class $\{\gamma_{p,hj}(0,t)\xi : t \in [0,\tau]\}$, it follows that

$$\begin{aligned}\sup_{t\in[0,\tau]}\left|\hat{C}_{n,hj}(0,t)-\tilde{C}_{n,hj}(0,t)\right| &\leqslant \sum_{p=1}^{2}\Bigg[\sup_{t\in[0,\tau]}\Big|\{\hat{W}_{hj}(t)-W_{hj}(t)\}\\ &\quad\times\sqrt{n}\mathbb{P}_n\{\hat{\gamma}_{p,hj}(0,t)-\gamma_{p,hj}(0,t)\}\xi\Big|\\ &\quad+V\sup_{t\in[0,\tau]}\left|\sqrt{n}\mathbb{P}_n\{\hat{\gamma}_{p,hj}(0,t)-\gamma_{p,hj}(0,t)\}\xi\right|\\ &\quad+O_p(1)\sup_{t\in[0,\tau]}\left|\hat{W}_{hj}(t)-W_{hj}(t)\right|\Bigg].\end{aligned}$$

The uniform consistency of $\hat{W}_{hj}(t)$ and the arguments used in the proof of part (ii) in

Theorem 2 lead to the conclusion that $\sup_{t\in[0,\tau]}\left|\hat{C}_{n,hj}(0,t)-\tilde{C}_{n,hj}(0,t)\right| = o_p(1)$ and, thus, $\hat{C}_{n,hj}(0,\cdot) \underset{\xi}{\overset{p}{\rightsquigarrow}} \mathbb{Z}_{hj}(0,\cdot)$ in $D[0,\tau]$.

By Theorem 2 and the bootstrap continuous mapping theorem it follows that

$$\sqrt{n}W_{hj}(\cdot)\{\hat{\Delta}^*_{n,hj}(0,\cdot)-\hat{\Delta}_{n,hj}(0,\cdot)\} \underset{U}{\overset{p}{\rightsquigarrow}} \mathbb{Z}_{hj}(0,\cdot) \quad in \;\; D[0,\tau].$$

By the (unconditional) multiplier central limit theorem (van der Vaart and Wellner, 1996) and a double application of the functional delta method, it follows that $\sqrt{n}\{\hat{P}^*_{n,phj}(0,\cdot)-\hat{P}_{n,phj}(0,\cdot)\}$, $p=1,2$, converge weakly (unconditionally) to tight mean zero Gaussian processes in $D[0,\tau]$. This result along with the uniform consistency of $\hat{W}_{hj}(t)$ lead to the conclusion that

$$\left|\sqrt{n}\{\hat{W}_{hj}(t)-W_{hj}(t)\}\{\hat{\Delta}^*_{n,hj}(0,t)-\hat{\Delta}_{n,hj}(0,t)\}\right|_{\infty} = o_p(1),$$

unconditionally. Consequently,

$$\sqrt{n}\hat{W}_{hj}(\cdot)\{\hat{\Delta}^*_{n,hj}(0,\cdot)-\hat{\Delta}_{n,hj}(0,\cdot)\} \underset{U}{\overset{p}{\rightsquigarrow}} \mathbb{Z}_{hj}(0,\cdot) \quad in \;\; D[0,\tau].$$

Part (ii) of Theorem 3 can be shown using similar arguments.

It has to be noted that the proposed Kolmogorov–Smirnov-type tests are consistent for any fixed alternative hypothesis. This follows from Theorem 3, the uniform consistency of the proposed estimators, the continuity of these tests in the differences $\hat{\Delta}_{n,hj}(s,t)$, $\hat{\Delta}_{n,j}(t)$, $\hat{\Delta}'_{n,hj}(s,t)$, and $\hat{\Delta}'_{n,j}(t)$, and Lemma 14.15 in van der Vaart (2000).

B.5 *Violation of condition C6*

It is possible that, in some applications, condition C6 is not satisfied. This happens when there are transient states with 0 probability of occupation in a subset of the observation time interval $[0,\tau]$. This is the case, for example, in situations where $P_{0,h}(0)=0$ for some transient state(s) h. Even though the consistency of the proposed estimators requires only conditions C1-C5, Theorems 2 and 3 additionally require condition C6. If condition C6 is

violated for some $h \in \mathcal{T}^c$, and in light of condition C4, it follows that

$$A_{0,hj}(t) = \int_{(0,t]\cap J_h} \frac{dPN_{\cdot,hj}(u)}{PY_{\cdot,h}(u)},$$

and

$$\hat{A}_{n,hj}(t) = \int_{(0,t]\cap J_h} \frac{d\mathbb{P}_n N_{\cdot,hj}(u)}{\mathbb{P}_n Y_{\cdot,h}(u)},$$

where $A_{0,hj}(t) = \hat{A}_{n,hj}(t) = 0$ if $t \in [0,t] \cap J_h^c$. In this case, the map $(F_1, F_2) \mapsto \int_{[0,t]\cap J_h} F_1^{-1} dF_2$ is Hadamard differentiable on the domain

$$\left\{ (F_1, F_2) : \inf_{t \in J_h} |F_1(t)| \geqslant \epsilon, \int_{J_h} |dF_2(t)| < \infty \right\}$$

for $\epsilon > 0$ and F_1^{-1} of bounded variation (Kosorok, 2008). Therefore, the same calculations to those used in the proof of Theorem 2 lead to the conclusion that

$$\begin{aligned} \sqrt{n}\{\hat{A}_{n,hj}(t) - A_{0,hj}(t)\} &= \sqrt{n}\mathbb{P}_n \int_{(0,t]\cap J_h} \frac{d\bar{M}_{hj}(u)}{PY_{\cdot,h}(u)} + o_p(1) \\ &= \sqrt{n}\mathbb{P}_n \phi_{hj}(t) + o_p(1), \quad t \in J_h, \end{aligned}$$

with the class $\{\phi_{hj}(t) : t \in J_h\}$ being P-Donsker. This means that $\sqrt{n}(\hat{A}_{n,hj} - A_{0,hj})$ converges weakly to a tight zero mean Gaussian process $\tilde{\mathbb{G}}_{3hj}$ in DJ_h with covariance function $P\phi_{hj}(t_1)\phi_{hj}(t_2)$, $t_1, t_2 \in J_h$, for $h \neq j$. The same arguments to those used in the proof of Theorem 2 can be used to show that this theorem holds for t restricted to $\cap_{h\in\mathcal{T}^c} J_h$. This means that inference about $P_{0,hj}(s,t)$, $h \neq j$, is possible for s and t in $\cap_{h\in\mathcal{T}^c} J_h$. From a practical standpoint one needs to restrict the time interval for confidence intervals/bands and hypothesis tests to a set such that there are at least some observations in all transient states.

B.6 *A remark on non-Markov processes*

As mentioned in the main manuscript, inference with non-Markov processes can be performed as indicated in Theorems 2 and 3, with the exception that the influence functions for the landmark versions of $\hat{P}_{n,hj}(s,t)$ and $\hat{P}'_{n,hj}(s,t)$ involve the modified processes $\tilde{N}_{im,lj}(t; h, s)$, $l \neq j$, and $\tilde{Y}_{im,l}(t; h, s)$, $l \in \mathcal{T}^c$. However, note that the influence functions of the estimators

$\hat{P}_{n,hj}(0,t)$, $\hat{P}'_{n,hj}(0,t)$, $\hat{P}_{n,j}(t)$, and $\hat{P}'_{n,j}(t)$ involve the quantities $P_{0,qj}(u,t)$ and $P'_{0,qj}(u,t)$, for $u > 0$. With non-Markov processes, these quantities are defined as the (q,j) element of the matrices $\prod_{(u,t]}\{\mathbf{I}_k + d\mathbf{A}_0(s)\}$ and $\prod_{(u,t]}\{\mathbf{I}_k + d\mathbf{A}'_0(s)\}$, respectively. The latter matrices are not necessarily equal to the true (conditional on the prior history) transition probability matrices under a non-Markov process. Nevertheless, the true influence functions depend on these matrices regardless of the Markov assumption. This is because, given the consistency of the estimators, the derivation of the influence functions in the proof of Theorem 2 (Web Appendix B.3) does not utilize the Markov assumption. The same phenomenon is observed for the independent observations setting (Glidden, 2002). Since these matrices are continuous in $\mathbf{A}_0(s)$ and $\mathbf{A}'_0(s)$ (Andersen et al., 2012), they can be consistently estimated by $\prod_{(u,t]}\{\mathbf{I}_k + d\hat{\mathbf{A}}_n(s)\}$ and $\prod_{(u,t]}\{\mathbf{I}_k + d\hat{\mathbf{A}}'_n(s)\}$. These can be used to estimate the influence functions of the estimators $\hat{P}_{n,hj}(0,t)$, $\hat{P}'_{n,hj}(0,t)$, $\hat{P}_{n,j}(t)$, and $\hat{P}'_{n,j}(t)$.

Web Appendix C: Test for Informative Cluster Size

For situations where cluster size is random, the proposed Kolmogorov–Smirnov tests can be applied for testing the null hypotheses $H_0 : P_{0,hj}(s,\cdot) = P'_{0,hj}(s,\cdot)$, for $s \in [0,\tau)$, or $H_0 : P_{0,j} = P'_{0,j}$, for the transition or state of the main scientific interest, respectively. Rejecting such a null hypotheses provides evidence for a violation of the non-informative cluster size assumption. The weighted difference functions for this hypothesis testing problem become $\hat{W}(t)\{\hat{P}_{n,hj}(s,t) - \hat{P}'_{n,hj}(s,t)\}$ and $\hat{W}(t)\{\hat{P}_{n,j}(t) - \hat{P}'_{n,j}(t)\}$. In light of Theorem 2 and the discussion in Section 2.4 of the main manuscript, these differences are asymptotically linear with influence functions $W(t)\{\gamma_{ihj}(s,t) - \gamma'_{ihj}(s,t)\}$ and $W(t)\{\psi_{ij}(t) - \psi'_{ij}(t)\}$ respectively, which both belong to P-Donsker classes. Furthermore, the cluster bootstrap versions of the weighted differences are

$$\hat{W}(t)[\{\hat{P}^*_{n,hj}(s,t) - \hat{P}'^*_{n,hj}(s,t)\} - \{\hat{P}_{n,hj}(s,t) - \hat{P}'_{n,hj}(s,t)\}]$$

and $\hat{W}(t)[\{\hat{P}^{*}_{n,j}(t)-\hat{P}'^{*}_{n,j}(t)\}-\{\hat{P}_{n,j}(t)-\hat{P}'_{n,j}(t)\}]$. With these (slight) modifications, a similar version of Theorem 3 holds for the aforementioned weighted differences and, thus, testing the null hypothesis $H_0 : P_{0,hj}(s,\cdot) = P'_{0,hj}(s,\cdot)$, for $s \in [0,\tau)$, or $H_0 : P_{0,j} = P'_{0,j}$ can be performed using the test statistics $\sup_{t\in[s,\tau]} |\hat{W}(t)\{\hat{P}_{n,hj}(s,t) - \hat{P}'_{n,hj}(s,t)\}|$ or $\sup_{t\in[0,\tau]} |\hat{W}(t)\{\hat{P}_{n,j}(t) - \hat{P}'_{n,j}(t)\}|$, as described in the last paragraph of Section 2.5 of the main manuscript.

Web Appendix D: Additional Simulation Results

This Web Appendix includes additional simulation results. The population-averaged probabilities of interest under the main simulation setup are depicted in Figure 1. Since, under the simulation setup the intensity for the transition $1 \to 2$ was lower for larger clusters, the population-averaged probabilities over the population of all cluster members $P_{0,2}(t)$ and $P_{0,12}(0.5,t)$ were lower compared to the corresponding ones for the population of typical cluster members $P'_{0,2}(t)$ and $P'_{0,12}(0.5,t)$ (Figure 1). This illustrates the fact that larger clusters have a larger influence on the population-averaged probabilities over the population of all cluster members. Web Appendix D.1 provides simulation results regarding the methods for the transition probabilities $P_{0,12}(0.5,t)$ and $P'_{0,12}(0.5,t)$ under right censoring. Web Appendix D.2 presents additional simulation results for the methods for state occupation and transition probabilities under both right censoring and left truncation. Finally, Web Appendix D.3 presents simulation results under a more variable cluster size and a very small number of clusters, in the presence of right censoring.

[Figure 1 about here.]

D.1 *Right Censoring*

Simulation results regarding the estimators of the population-averaged transition probabilities $P_{0,12}(0.5,t)$ and $P'_{0,12}(0.5,t)$ under right censoring are presented in Tables 1-4. Ignoring the within-cluster dependence was associated with underestimated standard errors

and poor coverage probabilities of the 95% pointwise confidence intervals and simultaneous confidence bands. This poor performance of the naïve methods for the transition probabilities $P_{0,12}(0.5,t)$ and $P'_{0,12}(0.5,t)$ was less pronounced compared to the case of the state occupation probabilities $P_{0,2}(t)$ and $P'_{0,2}(t)$. This is because estimation of $P_{0,12}(0.5,t)$ and $P'_{0,12}(0.5,t)$ under a non-Markov process only utilizes observations at state 1 at time $s = 0.5$ and, thus, it uses a smaller number of observations per cluster which leads to a less pronounced intracluster dependence issue. Also, the working-independence Aalen–Johansen estimator of $P'_{0,12}(0.5,t)$ exhibited some bias as a result of the informative cluster-size. On the contrary, the proposed methods performed well with the exception of somewhat lower coverage probabilities for the pointwise 95% confidence intervals and simultaneous confidence bands for the case of a very small number of clusters ($n = 20$) with only 5-15 observations per cluster.

[Table 1 about here.]

[Table 2 about here.]

[Table 3 about here.]

[Table 4 about here.]

D.2 *Right Censoring and Left Truncation*

Simulation results for the situation with both right censoring and left truncation are presented in Tables 5-12. As expected, the naïve methods which ignore the within-cluster dependence were associated with underestimated standard errors and poor coverage probabilities of the 95% pointwise confidence intervals and simultaneous confidence bands. The proposed methods performed well with the exception of lower coverage probabilities (reaching 91% in a few cases for the influence function-based intervals) for cases with a very small number of clusters ($n = 20$), and a small number of clusters ($n = 40$) with only 5-15 observations per cluster. This is attributed to the fact that with such small cluster sizes and under both

right censoring and left truncation, the amount of available information for estimating the parameters of interest was quite small. For such cases, the performance of the nonparametric cluster bootstrap was somewhat better compared to the influence function-based inference, indicating that the nonparametric cluster bootstrap may have a better performance for cases with a very small number of clusters. It is important to note that, even in such cases, the proposed methods outperformed their naïve counterparts.

[Table 5 about here.]

[Table 6 about here.]

[Table 7 about here.]

[Table 8 about here.]

[Table 9 about here.]

[Table 10 about here.]

[Table 11 about here.]

[Table 12 about here.]

D.3 *More Variable Cluster Size and Small Number of Clusters*

In order to evaluate the performance of the proposed methods under a larger cluster size variability and a very small number of clusters, we conducted an additional set of simulations experiments. Cluster size in these experiments was simulated from the discrete uniform distribution $\mathcal{U}[5, 200]$, while the number of clusters was either 15 or 20. These simulation results are presented in Tables 13-16. The naïve methods that ignore the within-cluster dependence provided seriously under-estimated standard errors and poor coverage probabilities, ranging from 31% to 67%. The performance of the proposed methods was still satisfactory with small bias, average standard error estimates close to the Monte Carlo standard deviation of the

estimates, and empirical type I error rates for the proposed Kolmogorov–Smirnov-type tests close to the 0.05 level in all cases. However, the empirical coverage probabilities for the 95% pointwise confidence intervals and simultaneous confidence bands were somewhat lower than the nominal level in some cases (reaching 91% in a few cases with only $n = 15$ clusters). The somewhat lower coverage probabilities are attributed to the very small numbers of clusters.

[Table 13 about here.]

[Table 14 about here.]

[Table 15 about here.]

[Table 16 about here.]

References

Aalen, O. O. and Johansen, S. (1978). An empirical transition matrix for non-homogeneous markov chains based on censored observations. *Scandinavian Journal of Statistics* **5,** 141–150.

Andersen, P. K., Borgan, O., Gill, R. D., and Keiding, N. (2012). *Statistical models based on counting processes.* Springer Science & Business Media.

Glidden, D. V. (2002). Robust inference for event probabilities with non-markov event data. *Biometrics* **58,** 361–368.

Kosorok, M. R. (2008). *Introduction to empirical processes and semiparametric inference.* Springer.

van der Vaart, A. W. (2000). *Asymptotic statistics.* Cambridge University Press.

van der Vaart, A. W. and Wellner, J. A. (1996). *Weak convergence and empirical processes with applications to Statistics.* Springer.

Received October 2004. *Revised February* 2005.

Accepted March 2005.

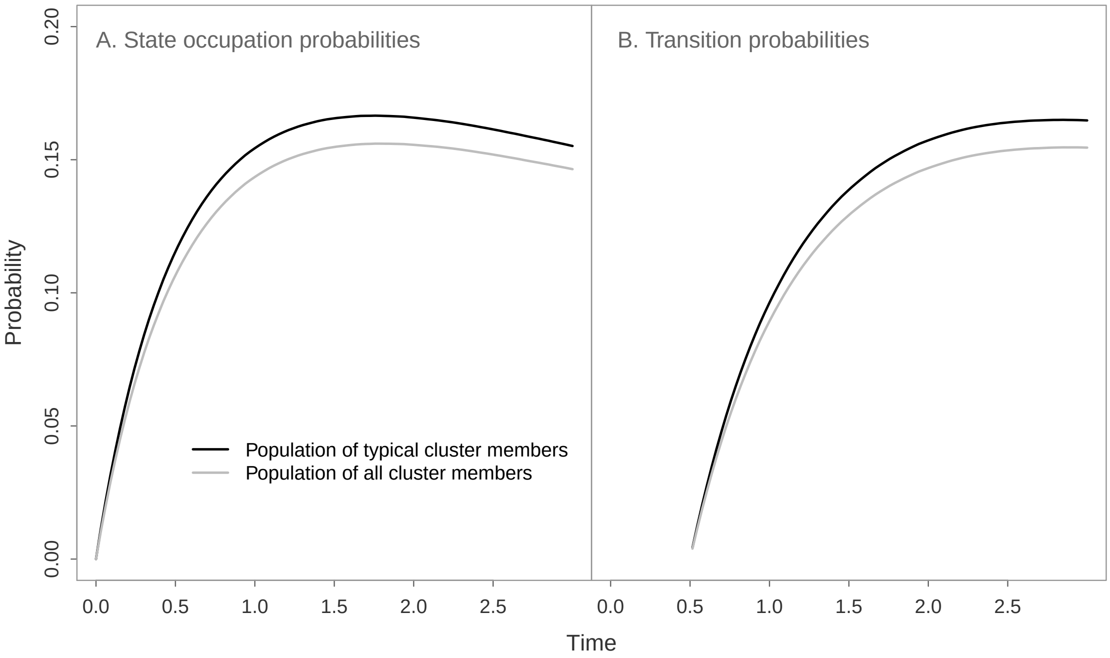

Figure 1: True population-averaged state occupation (A) and transition probabilities (B) under the basic simulation scenario.

Table 1: Simulation results for the analysis of $P_{0,12}(0.5, \tau_{0.4})$ and $P'_{0,12}(0.5, \tau_{0.4})$, where $\tau_{0.4}$ is the 40th percentile of the follow-up time conditional on survival at $t = 0.5$, based on the standard approach which ignores the within-cluster dependence (naïve) and the proposed method with i) the influence function-based variance estimator (IF) and ii) the nonparametric cluster bootstrap (CB). Results under right censoring. (n: number of clusters; F_M: discrete uniform distribution of the cluster size; *: $\times 10^2$; MCSD: Monte Carlo standard deviation of the estimates; ASE: average estimated standard error; CP: coverage probability).

			$P_{0,12}(0.5, \tau_{0.4})$				$P'_{0,12}(0.5, \tau_{0.4})$			
n	F_M	Method	Bias*	MCSD*	ASE*	CP	Bias*	MCSD*	ASE*	CP
20	$\mathcal{U}[5, 15]$	Naïve	0.296	3.603	3.065	0.913	-0.540	3.603	3.065	0.896
		IF	0.296	3.603	3.349	0.930	0.242	3.956	3.665	0.923
		CB	0.296	3.603	3.407	0.934	0.242	3.956	3.703	0.927
	$\mathcal{U}[10, 30]$	Naïve	0.184	2.653	2.158	0.897	-0.621	2.653	2.158	0.886
		IF	0.184	2.653	2.632	0.949	0.199	2.914	2.874	0.941
		CB	0.184	2.653	2.669	0.948	0.199	2.914	2.896	0.947
40	$\mathcal{U}[5, 15]$	Naïve	-0.048	2.389	2.142	0.924	-0.884	2.389	2.142	0.904
		IF	-0.048	2.389	2.370	0.945	-0.033	2.615	2.623	0.945
		CB	-0.048	2.389	2.387	0.945	-0.033	2.615	2.633	0.942
	$\mathcal{U}[10, 30]$	Naïve	0.073	1.874	1.518	0.888	-0.732	1.874	1.518	0.869
		IF	0.073	1.874	1.880	0.945	0.035	2.010	2.043	0.941
		CB	0.073	1.874	1.892	0.945	0.035	2.010	2.050	0.939
80	$\mathcal{U}[5, 15]$	Naïve	-0.017	1.729	1.517	0.910	-0.853	1.729	1.517	0.878
		IF	-0.017	1.729	1.699	0.941	-0.037	1.886	1.882	0.941
		CB	-0.017	1.729	1.705	0.939	-0.037	1.886	1.886	0.940
	$\mathcal{U}[10, 30]$	Naïve	0.092	1.389	1.076	0.871	-0.713	1.389	1.076	0.825
		IF	0.092	1.389	1.349	0.941	0.055	1.501	1.472	0.942
		CB	0.092	1.389	1.354	0.942	0.055	1.501	1.476	0.942

Table 2: Simulation results for the analysis of $P_{0,12}(0.5, \tau_{0.6})$ and $P'_{0,12}(0.5, \tau_{0.6})$, where $\tau_{0.6}$ is the 60th percentile of the follow-up time conditional on survival at $t = 0.5$, based on the standard approach which ignores the within-cluster dependence (naïve) and the proposed method with i) the influence function-based variance estimator (IF) and ii) the nonparametric cluster bootstrap (CB). Results under right censoring. (n: number of clusters; F_M: discrete uniform distribution of the cluster size; *: $\times 10^2$; MCSD: Monte Carlo standard deviation of the estimates; ASE: average estimated standard error; CP: coverage probability).

			$P_{0,12}(0.5, \tau_{0.6})$				$P'_{0,12}(0.5, \tau_{0.6})$			
n	F_M	Method	Bias*	MCSD*	ASE*	CP	Bias*	MCSD*	ASE*	CP
20	$\mathcal{U}[5, 15]$	Naïve	0.403	4.265	3.672	0.913	-0.591	4.265	3.672	0.909
		IF	0.403	4.265	3.919	0.916	0.339	4.717	4.264	0.915
		CB	0.403	4.265	3.989	0.924	0.339	4.717	4.314	0.918
	$\mathcal{U}[10, 30]$	Naïve	0.147	3.184	2.574	0.884	-0.807	3.184	2.574	0.872
		IF	0.147	3.184	3.037	0.927	0.103	3.436	3.303	0.933
		CB	0.147	3.184	3.076	0.933	0.103	3.436	3.327	0.933
40	$\mathcal{U}[5, 15]$	Naïve	0.039	2.774	2.573	0.934	-0.954	2.774	2.573	0.921
		IF	0.039	2.774	2.801	0.950	0.059	3.077	3.086	0.945
		CB	0.039	2.774	2.824	0.949	0.059	3.077	3.099	0.947
	$\mathcal{U}[10, 30]$	Naïve	0.026	2.218	1.814	0.896	-0.928	2.218	1.814	0.853
		IF	0.026	2.218	2.175	0.933	-0.002	2.409	2.362	0.932
		CB	0.026	2.218	2.189	0.936	-0.002	2.409	2.368	0.935
80	$\mathcal{U}[5, 15]$	Naïve	-0.001	2.019	1.815	0.922	-0.994	2.019	1.815	0.896
		IF	-0.001	2.019	1.989	0.937	-0.008	2.214	2.203	0.949
		CB	-0.001	2.019	1.995	0.938	-0.008	2.214	2.208	0.946
	$\mathcal{U}[10, 30]$	Naïve	0.082	1.572	1.287	0.889	-0.871	1.572	1.287	0.839
		IF	0.082	1.572	1.560	0.952	0.044	1.716	1.700	0.942
		CB	0.082	1.572	1.565	0.953	0.044	1.716	1.703	0.942

Table 3: Simulation results regarding the coverage probabilities of the 95% simultaneous confidence bands for $P_{0,12}(0.5,\cdot)$ and $P'_{0,12}(0.5,\cdot)$ based on the standard method that ignores the within-cluster dependence (naïve) and the proposed method with i) the estimated processes $\hat{B}_{n,12}(0.5,\cdot)$ and $\hat{B}'_{n,12}(0.5,\cdot)$ (IF) and ii) the nonparametric cluster bootstrap (CB). Results under right censoring. (n: number of clusters; F_M: discrete uniform distribution of the cluster size).

		$P_{0,12}(0.5,\cdot)$			$P'_{0,12}(0.5,\cdot)$		
n	F_M	Naïve	IF	CB	Naïve	IF	CB
20	$\mathcal{U}[5,15]$	0.909	0.931	0.942	0.890	0.911	0.927
	$\mathcal{U}[10,30]$	0.880	0.935	0.943	0.855	0.928	0.943
40	$\mathcal{U}[5,15]$	0.907	0.943	0.946	0.881	0.937	0.941
	$\mathcal{U}[10,30]$	0.887	0.945	0.952	0.853	0.946	0.951
80	$\mathcal{U}[5,15]$	0.915	0.941	0.946	0.870	0.940	0.946
	$\mathcal{U}[10,30]$	0.892	0.941	0.944	0.833	0.946	0.945

Table 4: Simulation results regarding the empirical type I error (H_0) and the empirical power (H_1) of the proposed two-sample Kolmogorov–Smirnov-type tests for $H_0 : P_{0,112}(0.5,\cdot) = P_{0,212}(0.5,\cdot)$ and $H_0 : P'_{0,112}(0.5,\cdot) = P'_{0,212}(0.5,\cdot)$ at the $\alpha = 0.05$ level. Significance levels were calculated based on either the estimated processes $\hat{C}_{n,12}(0.5,\cdot)$ and $\hat{C}'_{n,12}(0.5,\cdot)$ (IF) or the nonparametric cluster bootstrap (CB). Results under right censoring. (n: number of clusters; F_M: distribution of the cluster size).

		$P_{0,p12}(0.5,\cdot)$, $p = 1,2$				$P'_{0,p12}(0.5,\cdot)$, $p = 1,2$			
		H_0		H_1		H_0		H_1	
n	F_M	IF	CB	IF	CB	IF	CB	IF	CB
20	$\mathcal{U}[5,15]$	0.039	0.038	0.125	0.106	0.041	0.042	0.111	0.103
	$\mathcal{U}[10,30]$	0.036	0.029	0.197	0.169	0.040	0.034	0.172	0.150
40	$\mathcal{U}[5,15]$	0.046	0.037	0.217	0.206	0.038	0.032	0.187	0.181
	$\mathcal{U}[10,30]$	0.047	0.041	0.389	0.363	0.046	0.044	0.343	0.321
80	$\mathcal{U}[5,15]$	0.063	0.061	0.424	0.405	0.068	0.061	0.359	0.353
	$\mathcal{U}[10,30]$	0.049	0.046	0.729	0.714	0.051	0.046	0.658	0.656

Table 5: Simulation results for the analysis of $P_{0,2}(\tau_{0.4})$ and $P'_{0,2}(\tau_{0.4})$, where $\tau_{0.4}$ is the 40th percentile of the follow-up time, based on the standard approach which ignores the within-cluster dependence (naïve) and the proposed method with i) the influence function-based variance estimator (IF) and ii) the nonparametric cluster bootstrap (CB). Results under both right censoring and left truncation. (n: number of clusters; F_M: discrete uniform distribution of the cluster size; *: $\times 10^2$; MCSD: Monte Carlo standard deviation of the estimates; ASE: average estimated standard error; CP: coverage probability).

			$P_{0,2}(\tau_{0.4})$				$P'_{0,2}(\tau_{0.4})$			
n	F_M	Method	Bias*	MCSD*	ASE*	CP	Bias*	MCSD*	ASE*	CP
20	$\mathcal{U}[5,15]$	Naïve	0.270	3.823	3.332	0.926	-0.795	3.823	3.332	0.908
		IF	0.270	3.823	3.635	0.934	0.245	4.194	4.001	0.932
		CB	0.270	3.823	3.663	0.937	0.245	4.194	4.008	0.935
	$\mathcal{U}[10,30]$	Naïve	0.041	2.926	2.352	0.884	-1.024	2.926	2.352	0.865
		IF	0.041	2.926	2.839	0.929	-0.024	3.206	3.079	0.924
		CB	0.041	2.926	2.858	0.929	-0.024	3.206	3.084	0.923
40	$\mathcal{U}[5,15]$	Naïve	-0.068	2.674	2.339	0.917	-1.133	2.674	2.339	0.887
		IF	-0.068	2.674	2.579	0.932	-0.118	2.966	2.839	0.942
		CB	-0.068	2.674	2.590	0.936	-0.118	2.966	2.844	0.944
	$\mathcal{U}[10,30]$	Naïve	0.030	2.118	1.664	0.890	-1.035	2.118	1.664	0.827
		IF	0.030	2.118	2.045	0.940	-0.027	2.314	2.226	0.940
		CB	0.030	2.118	2.050	0.941	-0.027	2.314	2.225	0.937
80	$\mathcal{U}[5,15]$	Naïve	0.011	1.857	1.663	0.916	-1.054	1.857	1.663	0.864
		IF	0.011	1.857	1.854	0.941	-0.039	2.060	2.049	0.937
		CB	0.011	1.857	1.855	0.943	-0.039	2.060	2.047	0.938
	$\mathcal{U}[10,30]$	Naïve	-0.097	1.442	1.174	0.889	-1.162	1.442	1.174	0.774
		IF	-0.097	1.442	1.457	0.955	-0.164	1.567	1.595	0.952
		CB	-0.097	1.442	1.458	0.955	-0.164	1.567	1.594	0.954

Table 6: Simulation results for the analysis of $P_{0,2}(\tau_{0.6})$ and $P'_{0,2}(\tau_{0.6})$, where $\tau_{0.6}$ is the 60th percentile of the follow-up time, based on the standard approach which ignores the within-cluster dependence (naïve) and the proposed method with i) the influence function-based variance estimator (IF) and ii) the nonparametric cluster bootstrap (CB). Results under both right censoring and left truncation. (n: number of clusters; F_M: discrete uniform distribution of the cluster size; *: $\times 10^2$; MCSD: Monte Carlo standard deviation of the estimates; ASE: average estimated standard error; CP: coverage probability).

			$P_{0,2}(\tau_{0.6})$				$P'_{0,2}(\tau_{0.6})$			
n	F_M	Method	Bias*	MCSD*	ASE*	CP	Bias*	MCSD*	ASE*	CP
20	$\mathcal{U}[5,15]$	Naïve	0.144	4.229	3.784	0.928	-0.940	4.229	3.784	0.920
		IF	0.144	4.229	3.988	0.932	0.082	4.621	4.366	0.932
		CB	0.144	4.229	4.036	0.937	0.082	4.621	4.393	0.933
	$\mathcal{U}[10,30]$	Naïve	0.037	3.212	2.681	0.909	-1.047	3.212	2.681	0.871
		IF	0.037	3.212	3.080	0.931	-0.029	3.505	3.354	0.924
		CB	0.037	3.212	3.106	0.934	-0.029	3.505	3.365	0.929
40	$\mathcal{U}[5,15]$	Naïve	0.010	2.973	2.676	0.925	-1.074	2.973	2.676	0.911
		IF	0.010	2.973	2.862	0.935	-0.060	3.267	3.125	0.926
		CB	0.010	2.973	2.882	0.938	-0.060	3.267	3.139	0.929
	$\mathcal{U}[10,30]$	Naïve	0.110	2.253	1.902	0.901	-0.974	2.253	1.902	0.874
		IF	0.110	2.253	2.216	0.943	0.057	2.460	2.411	0.941
		CB	0.110	2.253	2.223	0.944	0.057	2.460	2.412	0.942
80	$\mathcal{U}[5,15]$	Naïve	0.111	1.993	1.902	0.934	-0.973	1.993	1.902	0.904
		IF	0.111	1.993	2.062	0.949	0.070	2.219	2.263	0.945
		CB	0.111	1.993	2.066	0.951	0.070	2.219	2.264	0.948
	$\mathcal{U}[10,30]$	Naïve	-0.086	1.543	1.339	0.904	-1.169	1.543	1.339	0.820
		IF	-0.086	1.543	1.576	0.953	-0.143	1.687	1.725	0.957
		CB	-0.086	1.543	1.577	0.955	-0.143	1.687	1.724	0.960

Table 7: Simulation results regarding the coverage probabilities of the 95% simultaneous confidence bands for $P_{0,2}(\cdot)$ and $P'_{0,2}(\cdot)$ based on the standard method that ignores the within-cluster dependence (naïve) and the proposed method with i) the estimated processes $\hat{B}_{n,2}$ and $\hat{B}'_{n,2}$ (IF) and ii) the nonparametric cluster bootstrap (CB). Results under both right censoring and left truncation. (n: number of clusters; F_M: discrete uniform distribution of the cluster size).

		$P_{0,2}(\cdot)$			$P'_{0,2}(\cdot)$		
n	F_M	Naïve	IF	CB	Naïve	IF	CB
20	$\mathcal{U}[5,15]$	0.884	0.933	0.945	0.869	0.928	0.931
	$\mathcal{U}[10,30]$	0.838	0.920	0.922	0.814	0.916	0.916
40	$\mathcal{U}[5,15]$	0.872	0.915	0.928	0.839	0.916	0.917
	$\mathcal{U}[10,30]$	0.848	0.938	0.943	0.794	0.939	0.942
80	$\mathcal{U}[5,15]$	0.906	0.948	0.950	0.848	0.950	0.949
	$\mathcal{U}[10,30]$	0.850	0.946	0.955	0.740	0.954	0.954

Table 8: Simulation results regarding the empirical type I error (H_0) and the empirical power (H_1) of the proposed two-sample Kolmogorov–Smirnov-type tests for $H_0 : P_{0,12}(\cdot) = P_{0,22}(\cdot)$ and $H_0 : P'_{0,12}(\cdot) = P'_{0,22}(\cdot)$ at the $\alpha = 0.05$ level. Significance levels were calculated based on either the estimated processes $\hat{C}_{n,2}$ and $\hat{C}'_{n,2}$ (IF) or the nonparametric cluster bootstrap (CB). Results under both right censoring and left truncation. (n: number of clusters; F_M: distribution of the cluster size).

		$P_{0,p2}(\cdot)$, $p = 1, 2$				$P'_{0,p2}(\cdot)$, $p = 1, 2$			
		H_0		H_1		H_0		H_1	
n	F_M	IF	CB	IF	CB	IF	CB	IF	CB
20	$\mathcal{U}[5, 15]$	0.051	0.048	0.249	0.240	0.051	0.044	0.245	0.246
	$\mathcal{U}[10, 30]$	0.053	0.049	0.472	0.465	0.054	0.055	0.430	0.448
40	$\mathcal{U}[5, 15]$	0.051	0.049	0.455	0.455	0.043	0.046	0.415	0.410
	$\mathcal{U}[10, 30]$	0.053	0.051	0.724	0.724	0.044	0.045	0.687	0.685
80	$\mathcal{U}[5, 15]$	0.055	0.048	0.738	0.728	0.055	0.049	0.695	0.686
	$\mathcal{U}[10, 30]$	0.047	0.044	0.962	0.961	0.047	0.048	0.941	0.941

Table 9: Simulation results for the analysis of $P_{0,12}(0.5, \tau_{0.4})$ and $P'_{0,12}(0.5, \tau_{0.4})$, where $\tau_{0.4}$ is the 40th percentile of the follow-up time conditional on survival at $t = 0.5$, based on the standard approach which ignores the within-cluster dependence (naïve) and the proposed method with i) the influence function-based variance estimator (IF) and ii) the nonparametric cluster bootstrap (CB). Results under both right censoring and left truncation. (n: number of clusters; F_M: discrete uniform distribution of the cluster size; *: $\times 10^2$; MCSD: Monte Carlo standard deviation of the estimates; ASE: average estimated standard error; CP: coverage probability).

			$P_{0,12}(0.5, \tau_{0.4})$				$P'_{0,12}(0.5, \tau_{0.4})$			
n	F_M	Method	Bias*	MCSD*	ASE*	CP	Bias*	MCSD*	ASE*	CP
20	$\mathcal{U}[5, 15]$	Naïve	0.326	3.852	3.493	0.941	-0.542	3.852	3.493	0.930
		IF	0.326	3.852	3.728	0.943	0.327	4.346	4.112	0.942
		CB	0.326	3.852	3.805	0.948	0.327	4.346	4.168	0.944
	$\mathcal{U}[10, 30]$	Naïve	0.220	3.094	2.471	0.897	-0.617	3.094	2.471	0.865
		IF	0.220	3.094	2.864	0.930	0.187	3.376	3.121	0.922
		CB	0.220	3.094	2.906	0.933	0.187	3.376	3.148	0.923
40	$\mathcal{U}[5, 15]$	Naïve	0.024	2.723	2.448	0.924	-0.844	2.723	2.448	0.918
		IF	0.024	2.723	2.669	0.947	-0.023	3.022	2.935	0.939
		CB	0.024	2.723	2.691	0.947	-0.023	3.022	2.949	0.941
	$\mathcal{U}[10, 30]$	Naïve	0.236	2.102	1.751	0.903	-0.600	2.102	1.751	0.880
		IF	0.236	2.102	2.078	0.944	0.240	2.316	2.268	0.937
		CB	0.236	2.102	2.093	0.946	0.240	2.316	2.278	0.941
80	$\mathcal{U}[5, 15]$	Naïve	0.021	1.902	1.739	0.921	-0.847	1.902	1.739	0.898
		IF	0.021	1.902	1.902	0.949	-0.017	2.095	2.100	0.945
		CB	0.021	1.902	1.913	0.949	-0.017	2.095	2.109	0.944
	$\mathcal{U}[10, 30]$	Naïve	0.055	1.515	1.228	0.890	-0.782	1.515	1.228	0.828
		IF	0.055	1.515	1.477	0.942	0.048	1.669	1.621	0.940
		CB	0.055	1.515	1.483	0.943	0.048	1.669	1.625	0.944

Table 10: Simulation results for the analysis of $P_{0,12}(0.5, \tau_{0.6})$ and $P'_{0,12}(0.5, \tau_{0.6})$, where $\tau_{0.6}$ is the 60th percentile of the follow-up time conditional on survival at $t = 0.5$, based on the standard approach which ignores the within-cluster dependence (naïve) and the proposed method with i) the influence function-based variance estimator (IF) and ii) the nonparametric cluster bootstrap (CB). Results under both right censoring and left truncation. (n: number of clusters; F_M: discrete uniform distribution of the cluster size; *: $\times 10^2$; MCSD: Monte Carlo standard deviation of the estimates; ASE: average estimated standard error; CP: coverage probability).

			$P_{0,12}(0.5, \tau_{0.6})$				$P'_{0,12}(0.5, \tau_{0.6})$			
n	F_M	Method	Bias*	MCSD*	ASE*	CP	Bias*	MCSD*	ASE*	CP
20	$\mathcal{U}[5, 15]$	Naïve	0.342	4.514	4.138	0.934	-0.662	4.514	4.138	0.927
		IF	0.342	4.514	4.362	0.927	0.356	4.965	4.817	0.940
		CB	0.342	4.514	4.466	0.935	0.356	4.965	4.900	0.943
	$\mathcal{U}[10, 30]$	Naïve	0.219	3.503	2.931	0.901	-0.747	3.503	2.931	0.893
		IF	0.219	3.503	3.292	0.926	0.175	3.857	3.566	0.916
		CB	0.219	3.503	3.340	0.929	0.175	3.857	3.599	0.916
40	$\mathcal{U}[5, 15]$	Naïve	0.070	3.235	2.904	0.938	-0.935	3.235	2.904	0.912
		IF	0.070	3.235	3.107	0.952	0.037	3.590	3.419	0.942
		CB	0.070	3.235	3.133	0.957	0.037	3.590	3.436	0.946
	$\mathcal{U}[10, 30]$	Naïve	0.342	2.523	2.079	0.899	-0.624	2.523	2.079	0.900
		IF	0.342	2.523	2.390	0.929	0.307	2.766	2.593	0.932
		CB	0.342	2.523	2.405	0.931	0.307	2.766	2.602	0.932
80	$\mathcal{U}[5, 15]$	Naïve	0.035	2.193	2.064	0.940	-0.969	2.193	2.064	0.898
		IF	0.035	2.193	2.215	0.952	-0.021	2.424	2.441	0.949
		CB	0.035	2.193	2.224	0.954	-0.021	2.424	2.446	0.945
	$\mathcal{U}[10, 30]$	Naïve	0.022	1.773	1.452	0.884	-0.945	1.773	1.452	0.843
		IF	0.022	1.773	1.691	0.944	-0.002	1.943	1.847	0.931
		CB	0.022	1.773	1.695	0.942	-0.002	1.943	1.849	0.933

Table 11: Simulation results regarding the coverage probabilities of the 95% simultaneous confidence bands for $P_{0,12}(0.5,\cdot)$ and $P'_{0,12}(0.5,\cdot)$ based on the standard method that ignores the within-cluster dependence (naïve) and the proposed method with i) the estimated processes $\hat{B}_{n,12}(0.5,\cdot)$ and $\hat{B}'_{n,12}(0.5,\cdot)$ (IF) and ii) the nonparametric cluster bootstrap (CB). Results under both right censoring and left truncation. (n: number of clusters; F_M: discrete uniform distribution of the cluster size).

		$P_{0,12}(0.5,\cdot)$			$P'_{0,12}(0.5,\cdot)$		
n	F_M	Naïve	IF	CB	Naïve	IF	CB
20	$\mathcal{U}[5,15]$	0.892	0.916	0.941	0.882	0.906	0.928
	$\mathcal{U}[10,30]$	0.881	0.914	0.935	0.869	0.909	0.917
40	$\mathcal{U}[5,15]$	0.903	0.931	0.937	0.879	0.923	0.936
	$\mathcal{U}[10,30]$	0.903	0.941	0.950	0.866	0.933	0.940
80	$\mathcal{U}[5,15]$	0.919	0.939	0.944	0.875	0.935	0.936
	$\mathcal{U}[10,30]$	0.906	0.949	0.952	0.846	0.941	0.942

Table 12: Simulation results regarding the empirical type I error (H_0) and the empirical power (H_1) of the proposed two-sample Kolmogorov–Smirnov-type tests for $H_0 : P_{0,112}(0.5, \cdot) = P_{0,212}(0.5, \cdot)$ and $H_0 : P'_{0,112}(0.5, \cdot) = P'_{0,212}(0.5, \cdot)$ at the $\alpha = 0.05$ level. Significance levels were calculated based on either the estimated processes $\hat{C}_{n,12}(0.5, \cdot)$ and $\hat{C}'_{n,12}(0.5, \cdot)$ (IF) or the nonparametric cluster bootstrap (CB). Results under both right censoring and left truncation. (n: number of clusters; F_M: distribution of the cluster size).

		$P_{0,p12}(0.5, \cdot)$, $p = 1, 2$				$P'_{0,p12}(0.5, \cdot)$, $p = 1, 2$			
		H_0		H_1		H_0		H_1	
n	F_M	IF	CB	IF	CB	IF	CB	IF	CB
20	$\mathcal{U}[5, 15]$	0.037	0.037	0.139	0.124	0.037	0.040	0.129	0.111
	$\mathcal{U}[10, 30]$	0.044	0.044	0.235	0.200	0.049	0.043	0.195	0.192
40	$\mathcal{U}[5, 15]$	0.062	0.052	0.239	0.219	0.059	0.057	0.234	0.216
	$\mathcal{U}[10, 30]$	0.049	0.042	0.410	0.385	0.041	0.044	0.374	0.366
80	$\mathcal{U}[5, 15]$	0.060	0.048	0.379	0.407	0.052	0.049	0.332	0.368
	$\mathcal{U}[10, 30]$	0.054	0.048	0.708	0.700	0.054	0.056	0.661	0.650

Table 13: Simulation results for the analysis of $P_{0,2}(\tau_{0.4})$ and $P'_{0,2}(\tau_{0.4})$, where $\tau_{0.4}$ is the 40th percentile of the follow-up time, based on the standard approach which ignores the within-cluster dependence (naïve) and the proposed method with i) the influence function-based variance estimator (IF) and ii) the nonparametric cluster bootstrap (CB). Results under right censoring, a large variability of cluster size M_i, and a small number of clusters. (n: number of clusters; F_M: discrete uniform distribution of the cluster size; *: $\times 10^2$; MCSD: Monte Carlo standard deviation of the estimates; ASE: average estimated standard error; CP: coverage probability).

			$P_{0,2}(\tau_{0.4})$				$P'_{0,2}(\tau_{0.4})$			
n	F_M	Method	Bias*	MCSD*	ASE*	CP	Bias*	MCSD*	ASE*	CP
15	$\mathcal{U}[5, 200]$	Naïve	-0.040	2.216	0.917	0.590	-1.876	2.216	0.917	0.440
		IF	-0.040	2.216	2.092	0.918	-0.143	2.504	2.484	0.934
		CB	-0.040	2.216	2.131	0.922	-0.143	2.504	2.486	0.931
20		Naïve	0.040	1.957	0.798	0.587	-1.797	1.957	0.798	0.425
		IF	0.040	1.957	1.867	0.925	0.041	2.332	2.187	0.926
		CB	0.040	1.957	1.894	0.927	0.041	2.332	2.190	0.924

Table 14: Simulation results for the analysis of $P_{0,2}(\tau_{0.6})$ and $P'_{0,2}(\tau_{0.6})$, where $\tau_{0.6}$ is the 60th percentile of the follow-up time, based on the standard approach which ignores the within-cluster dependence (naïve) and the proposed method with i) the influence function-based variance estimator (IF) and ii) the nonparametric cluster bootstrap (CB). Results under right censoring, a small number of clusters, and a large variability of cluster size M_i. (n: number of clusters; F_M: discrete uniform distribution of the cluster size; *: $\times 10^2$; MCSD: Monte Carlo standard deviation of the estimates; ASE: average estimated standard error; CP: coverage probability).

			$P_{0,2}(\tau_{0.6})$				$P'_{0,2}(\tau_{0.6})$			
n	F_M	Method	Bias*	MCSD*	ASE*	CP	Bias*	MCSD*	ASE*	CP
15	$\mathcal{U}[5,200]$	Naïve	-0.069	2.297	1.062	0.637	-1.991	2.297	1.062	0.507
		IF	-0.069	2.297	2.102	0.913	-0.201	2.674	2.527	0.934
		CB	-0.069	2.297	2.143	0.922	-0.201	2.674	2.529	0.935
20		Naïve	-0.003	1.939	0.924	0.668	-1.925	1.939	0.924	0.466
		IF	-0.003	1.939	1.875	0.931	-0.114	2.312	2.217	0.933
		CB	-0.003	1.939	1.902	0.935	-0.114	2.312	2.218	0.934

Table 15: Simulation results regarding the coverage probabilities of the 95% simultaneous confidence bands for $P_{0,2}(\cdot)$ and $P'_{0,2}(\cdot)$ based on the standard method that ignores the within-cluster dependence (naïve) and the proposed method with i) the estimated processes $\hat{B}_{n,2}$ and $\hat{B}'_{n,2}$ (IF) and ii) the nonparametric cluster bootstrap (CB). Results under right censoring, a small number of clusters, and a large variability of cluster size M_i. (n: number of clusters; F_M: discrete uniform distribution of the cluster size).

		$P_{0,2}(\cdot)$			$P'_{0,2}(\cdot)$		
n	F_M	Naïve	IF	CB	Naïve	IF	CB
15	$\mathcal{U}[5, 200]$	0.478	0.907	0.924	0.359	0.925	0.925
20		0.488	0.930	0.940	0.313	0.926	0.923

Table 16: Simulation results regarding the empirical type I error (H_0) and the empirical power (H_1) of the proposed two-sample Kolmogorov–Smirnov-type tests for $H_0 : P_{0,12}(\cdot) = P_{0,22}(\cdot)$ and $H_0 : P'_{0,12}(\cdot) = P'_{0,22}(\cdot)$ at the $\alpha = 0.05$ level. Significance levels were calculated based on either the estimated processes $\hat{C}_{n,2}$ and $\hat{C}'_{n,2}$ (IF) or the nonparametric cluster bootstrap (CB). Results under right censoring, a small number of clusters, and a large variability of cluster size M_i. (n: number of clusters; F_M: distribution of the cluster size).

		$P_{0,p2}(\cdot)$, $p = 1, 2$				$P'_{0,p2}(\cdot)$, $p = 1, 2$			
		H_0		H_1		H_0		H_1	
n	F_M	IF	CB	IF	CB	IF	CB	IF	CB
15	$\mathcal{U}[5, 200]$	0.056	0.052	0.981	0.975	0.038	0.041	0.844	0.848
20		0.064	0.052	0.997	0.994	0.053	0.060	0.917	0.914
