# Supplementary material for: Nonparametric analysis of nonhomogeneous multistate processes with clustered observations
Source: Biometrics. 2020 Jul 21;77(2):533–46. doi: 10.1111/biom.13327 (PMC7790918; doi:10.1111/biom.13327)
Supplement: Supplementary file 2 [file BIOM-77-533-s001.zip › README.docx]

**Instructions for the code code_msm_clust.R**

**Description**

This code provides functions to implement the non-parametric estimators for transition and state occupation probabilities with clustered data under the progressive illness-death model. It also provides 95% pointwise confidence intervals and simultaneous confidence bands. Additionally, it implements the Kolmogorov-Smirnov-type test for the two-sample comparison of transition and state occupation probabilities.

**Dependencies**

None.

**Main functions**

i) est_msm_cl: Nonparametric estimation and 95% confidence intervals for transition and state occupation probabilities under the progressive illness-death model based on clustered observations

ii) test_msm_cl: Nonparametric two-sample Kolmogorov-Smirnov-type tests transition and state occupation probabilities under the progressive illness-death model based on clustered observations

**Inputs for est_msm_cl**

data: A data frame with multiple records per individual containing the variables:

cid: The unique id for the clusters (e.g. clinic code)

id: The unique id for the individuals

t0: Starting time of the interval

t1: Stopping time of the interval

s0: The state at t0

s1: The state visited at t1

s: The time of the process in P(X(t)=j | X(s)=h). Note that in the progressive illness-death model if s=0, then P(X(t)=j | X(0)=0) = P(X(t)=j)

tau: Maximum time to be considered

from.state: The state h of the process in P(X(t)=j | X(s)=h)

to.state: The state j of the process in P(X(t)=j | X(s)=h)

repl: number of cluster bootstrap replications

weighted: Selects either the weighted (by cluster size) estimator if weighted = 1 or the unweighted estimator if weighted = 0

times: time points for the calculation of pointwise results

**Inputs for test_msm_cl**

data: A data frame with multiple records per individual containing the variables:

cid: The unique id for the clusters (e.g. clinic code)

id: The unique id for the individuals

t0: Starting time of the interval

t1: Stopping time of the interval

s0: The state at t0

s1: The state visited at t1

group: a binary 0/1 variable

s: The time of the process in P(X(t)=j | X(s)=h). Note that in the progressive illness-death model if s=0, then P(X(t)=j | X(0)=0) = P(X(t)=j)

tau: Maximum time to be considered

from.state: The state h of the process in P(X(t)=j | X(s)=h)

to.state: The state j of the process in P(X(t)=j | X(s)=h)

repl: number of cluster bootstrap replications

weighted: Selects either the weighted (by cluster size) estimator if weighted = 1 or the unweighted estimator if weighted = 0

group: the nave of the binary 0/1 grouping variable

**Examples**

setwd("C:/mydir")

source("code_msm_clust.R")

library(foreign)

data <- read.csv("example_data.csv")

The dataset “example_data.csv” contains clustered observations from the progressive illness-death model:

In the examples below we use 1000 cluster bootstrap replications.

Estimate $\hat{P}_{2}\left( t \right)=\hat{P}_{02}\left( 0,t \right)$, the state occupation probability of state 2, and provide estimates at times 0.5, 1, and 2:

P2 <- est_msm_cl(data=data, repl=1000, s=0, from.state=0, to.state=2, times=c(0.5,1,2))

Estimate $\hat{P}_{1}\left( t \right)=\hat{P}_{01}\left( 0,t \right)$, the state occupation probability of state 1, and provide estimates at times 0.5, 1, and 2:

P1 <- est_msm_cl(data=data, repl=1000, s=0, from.state=0, to.state=1, times=c(0.5,1,2))

Estimate the transition probability $\hat{P}_{12}\left( 0.5,t \right)$, and provide estimates at times 1 and 2:

P12.05 <- est_msm_cl(data=data, repl=1000, s=0.5, from.state=1, to.state=2, times=c(1,2))

Estimate the transition probability $\hat{P}_{01}\left( 0.5,t \right)$, and provide estimates at times 1 and 2:

P01.05 <- est_msm_cl(data=data, repl=1000, s=0.5, from.state=0, to.state=1, times=c(1,2))

Compare the state occupation probability $\hat{P}_{1}\left( t \right)$ between groups:

test_msm_cl(data=data, repl=1000, s=0, from.state=0, to.state=1, group="z")

Compare the transition probability $\hat{P}_{01}\left( 0.5,t \right)$ between groups:

test_msm_cl(data=data, repl=1000, s=0.5, from.state=0, to.state=1, group="z")
